# Computational Investigation of Montelukast and Its Structural Derivatives for Binding Affinity to Dopaminergic and Serotonergic Receptors: Insights from a Comprehensive Molecular Simulation

**DOI:** 10.3390/ph18040559

**Published:** 2025-04-10

**Authors:** Nasser Alotaiq, Doni Dermawan

**Affiliations:** 1 Health Sciences Research Center (HSRC), Imam Mohammad Ibn Saud Islamic University (IMSIU), Riyadh 13317, Saudi Arabia; 2 Department of Applied Biotechnology, Faculty of Chemistry, Warsaw University of Technology, 00-661 Warsaw, Poland; doni.dermawan.stud@pw.edu.pl

**Keywords:** 5-HT1A, D2 dopamine, molecular docking, molecular dynamics, montelukast, pharmacophore modeling

## Abstract

**Background/Objectives**: Montelukast (MLK), a leukotriene receptor antagonist, has been associated with neuropsychiatric side effects. This study aimed to rationally modify MLK’s structure to reduce these risks by optimizing its interactions with dopamine D2 (DRD2) and serotonin 5-HT1A receptors using computational molecular simulation techniques. **Methods**: A library of MLK derivatives was designed and screened using structural similarity analysis, molecular docking, molecular dynamics (MD) simulations, MM/PBSA binding free energy calculations, and ADME-Tox predictions. Structural similarity analysis, based on Tanimoto coefficient fingerprinting, compared MLK derivatives to known neuropsychiatric drugs. Docking was performed to assess initial receptor binding, followed by 100 ns MD simulations to evaluate binding stability. MM/PBSA calculations quantified binding affinities, while ADME-Tox profiling predicted pharmacokinetic and toxicity risks. **Results**: Several MLK derivatives showed enhanced DRD2 and 5-HT1A binding. MLK_MOD-42 and MLK_MOD-43 emerged as the most promising candidates, exhibiting MM/PBSA binding free energies of −31.92 ± 2.54 kcal/mol and −27.37 ± 2.22 kcal/mol for DRD2 and −30.22 ± 2.29 kcal/mol and −28.19 ± 2.14 kcal/mol for 5-HT1A, respectively. Structural similarity analysis confirmed that these derivatives share key pharmacophoric features with atypical antipsychotics and anxiolytics. However, off-target interactions were not assessed, which may influence their overall safety profile. ADME-Tox analysis predicted improved oral bioavailability and lower neurotoxicity risks. **Conclusions**: MLK_MOD-42 and MLK_MOD-43 exhibit optimized receptor interactions and enhanced pharmacokinetics, suggesting potential neuropsychiatric applications. However, their safety and efficacy remain to be validated through in vitro and in vivo studies. Until such validation is performed, these derivatives should be considered as promising candidates with optimized receptor binding rather than confirmed safer alternatives.

## 1. Introduction

Montelukast (MLK), a leukotriene receptor antagonist (LTRA), is commonly prescribed for asthma and allergic rhinitis due to its ability to reduce inflammation and bronchoconstriction [1,2]. However, increasing reports have linked Montelukast to neuropsychiatric adverse events (NPAEs), including anxiety, depression, sleep disturbances, and even suicidal ideation [3,4]. In response to these concerns, the U.S. Food and Drug Administration (FDA) issued a boxed warning in 2020, emphasizing the potential risks associated with Montelukast use, particularly in younger patients [5,6]. Despite these warnings, the precise molecular mechanisms responsible for Montelukast-induced neuropsychiatric effects remain poorly understood. Recent findings suggest that Montelukast is capable of crossing the blood–brain barrier (BBB), raising concerns about its unintended interactions with central nervous system (CNS) targets [7,8]. Among the neurotransmitter systems potentially affected, the dopaminergic (D2 dopamine receptor, DRD2) and serotonergic (5-HT1A receptor) pathways have been implicated in neuropsychiatric disorders [9,10]. DRD2 antagonism is a key mechanism of many antipsychotic drugs, while 5-HT1A receptor agonists are frequently used in the treatment of anxiety and depression [11,12]. However, it remains unclear whether Montelukast directly interacts with these receptors or if its modifications can enhance receptor selectivity without introducing unintended off-target interactions.

Although Montelukast has been associated with neuropsychiatric side effects, its exact interaction with dopaminergic and serotonergic receptors remains largely unexplored. Previous research has primarily focused on observational studies that document adverse effects without delving into the molecular mechanisms underlying these interactions [13,14]. There is currently a lack of molecular-level insights into Montelukast’s potential binding to DRD2 and 5-HT1A receptors, leaving its role in modulating these neurotransmitter systems speculative. Additionally, no prior studies have attempted to rationally modify Montelukast’s structure to optimize its affinity for DRD2 and 5-HT1A receptors. Standard antipsychotic drugs targeting DRD2, such as Haloperidol, and anxiolytics acting on 5-HT1A, such as Buspirone, have well-characterized binding profiles. However, Montelukast has not been systematically altered or compared to these standard drugs to determine whether structural modifications could yield beneficial pharmacological properties. More importantly, while optimizing receptor affinity is essential in drug repurposing, it does not directly correlate with improved safety or clinical efficacy. A comprehensive evaluation of potential off-target effects is necessary before making definitive claims regarding the therapeutic advantages of modified Montelukast derivatives.

The primary goal of this study was to rationally modify Montelukast’s structure to potentially reduce its neuropsychiatric risks by optimizing its interaction with DRD2 and 5-HT1A receptors using computational molecular simulation techniques. Through a series of in silico analyses, this research aimed to identify novel Montelukast derivatives that retained their therapeutic benefits while minimizing adverse effects on the CNS. To achieve this, a 3D structure modification strategy was employed to generate Montelukast derivatives that mimicked the binding characteristics of a standard DRD2 antagonist and a 5-HT1A agonist. Molecular docking simulations were conducted to evaluate the binding affinity and interaction patterns of the modified compounds with DRD2 and 5-HT1A receptors, with Haloperidol and Buspirone serving as reference drugs for comparison. Subsequently, molecular dynamics (MD) simulations were performed to assess the stability, conformational changes, and interaction dynamics of the ligand–receptor complexes over time. Additionally, pharmacophore modeling was used to identify key structural features responsible for DRD2 antagonism and 5-HT1A agonism, allowing for a comparison between Montelukast derivatives and standard reference drugs. Finally, in silico ADME and toxicity assessments were conducted to predict drug-likeness, BBB permeability, metabolism, and potential toxicity risks of the modified compounds.

However, it is important to note that these computational approaches primarily provide insights into binding affinity, pharmacokinetics, and predicted toxicity rather than definitive safety or efficacy data. As such, the modified Montelukast derivatives identified in this study should not be considered inherently safer or more effective until further experimental validation, including off-target screening and in vivo studies, is conducted. By ensuring favorable pharmacokinetics and potentially reduced neurotoxicity, this study aimed to develop optimized Montelukast derivatives with enhanced CNS selectivity and possible therapeutic efficacy. Thus, this research sought to bridge the existing knowledge gap by providing a molecular-level understanding of Montelukast’s interactions with DRD2 and 5-HT1A receptors. The findings from this study could serve as a foundation for further experimental validation and contribute to the development of novel therapeutic agents with hypothetically improved safety profiles for neuropsychiatric conditions.

## 2. Results

### 2.1. Modification Strategy and Similarity Analysis

**Figure 1:**
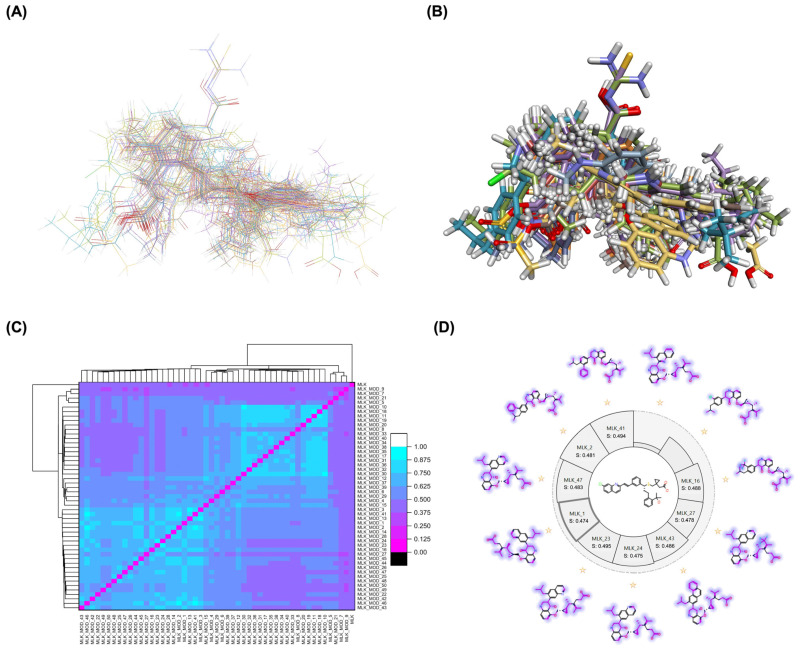
Structural alignment and similarity analysis of Montelukast and its modifications. (**A**) Superimposition of Montelukast modifications with the native structure (backbone representation). (**B**) A 3D superimposition of Montelukast modifications with the native structure. Different colors represent different Montelukast modifications, allowing visual comparison of structural variations. (**C**) Pairwise similarity correlation heatmap, depicting the structural similarity scores between Montelukast and its modified derivatives. Higher similarity scores indicate minimal structural deviation, while lower scores suggest significant modifications. (**D**) Similarity score distribution plot, providing a comparative analysis of the degree of structural resemblance between each modified derivative and the native Montelukast. Purple colors indicate the similarity of each modification compared to the native structure of Montelukast.

A comprehensive structural modification strategy was employed to explore the structure–activity relationship of Montelukast, aiming to enhance its pharmacokinetic and pharmacodynamic properties. The strategy encompassed a diverse set of chemical modifications, including aromatic substitutions, heterocyclic replacements, amide modifications, halogenation, carboxyl alterations, etherification, and side-chain modifications (Table 1). The specific derivatives presented in Table 1 were selected based on their most favorable docking results for both targeted receptors (DRD2 and 5-HT1A). These modifications were strategically designed to optimize Montelukast’s receptor binding affinity, metabolic stability, solubility, lipophilicity, and bioavailability. Following energy minimization, the modified structures were superimposed onto the native Montelukast backbone to evaluate structural conservation and deviation. The 3D superimposition analysis revealed that despite extensive modifications, the core structural framework of Montelukast remained largely intact, with key pharmacophoric features preserved. Given the broad scope of modifications, a flexible root-mean-square deviation (RMSD) threshold was applied to accommodate structural variations while ensuring alignment accuracy. Figure 1A,B illustrate this superimposition, with Figure 1A depicting the backbone alignment and Figure 1B providing a detailed 3D representation. The complete list of Montelukast modifications is available in Supplementary Data S1.

To further assess the degree of structural similarity, a pairwise similarity correlation heatmap (Figure 1C) was generated. The structural similarity between the modified derivatives and native Montelukast was found to be within the range of 0.25 to 0.50, which is considered low to moderate, indicating significant structural deviation. In contrast, inter-modification similarity values ranged from 0.625 to 1.00, suggesting that the modified derivatives retained significant structural resemblance to each other. Notably, MLK_MOD-2 (biphenyl substitution), MLK_MOD-24 (benzimidazole substitution), and MLK_MOD-43 (carbazole substitution) exhibited similarity values of 0.481, 0.475, and 0.486, respectively (Figure 1D), placing them at the upper range of similarity to Montelukast and suggesting partial structural conservation. These results indicate that while the modifications introduce distinct chemical properties, they maintain a reasonable level of structural conservation with the native molecule. The observed similarity trends suggest that certain modification strategies (such as heterocyclic replacements, halogenation, and fused ring systems) tend to introduce greater deviations from the native structure due to significant alterations in electronic properties and steric configurations. On the other hand, modifications involving functional group substitutions (e.g., amide, carboxyl, and hydroxyl modifications) generally exhibited higher similarity scores, reflecting minimal perturbations to the overall molecular framework. This balance between structural conservation and chemical diversification is critical for optimizing Montelukast derivatives while maintaining receptor compatibility.

Aromatic modifications, such as biphenyl (MLK_MOD-2) and fluorophenyl (MLK_MOD-3) substitutions, were introduced to enhance π-π stacking interactions with the target receptor. This strategy is particularly beneficial for improving binding affinity and receptor specificity, as increased aromatic surface area can facilitate stronger hydrophobic and van der Waals interactions. Similarly, heterocyclic replacements (e.g., pyridine in MLK_MOD-4 and thiophene in MLK_MOD-5) were designed to introduce polar functional groups that enhance hydrogen bonding interactions while maintaining a favorable balance of lipophilicity and metabolic stability. Amide modifications (e.g., methylation at amide nitrogen in MLK_MOD-8 and urea substitution in MLK_MOD-9) were specifically incorporated to fine-tune hydrogen bonding potential and receptor selectivity. These modifications can significantly influence protein–ligand interactions by stabilizing key binding interactions while also affecting drug metabolism and excretion profiles. Carboxyl modifications, such as carboxyl-to-amide (MLK_MOD-10) and carboxyl-to-ester (MLK_MOD-11) substitutions, were aimed at improving membrane permeability and lipophilicity, thereby enhancing oral bioavailability. Halogenation strategies (e.g., fluorine addition in MLK_MOD-13 and chlorine substitution in MLK_MOD-14) were explored to improve metabolic stability and enhance hydrophobic interactions. The incorporation of halogens often increases binding affinity by strengthening dipole-induced dipole interactions while also enhancing the compound’s resistance to enzymatic degradation. Similarly, fluorinated modifications (e.g., trifluoromethyl addition in MLK_MOD-16) were designed to improve BBB permeability, which is crucial for CNS-targeting applications. Hydroxylation (MLK_MOD-12), etherification (MLK_MOD-15), and sulfonylation (MLK_MOD-17) were applied to increase water solubility and hydrophilicity. While these modifications enhance aqueous solubility, they also introduce potential challenges related to plasma protein binding and renal clearance, requiring a careful balance between hydrophilic and lipophilic properties. The introduction of fused ring systems (e.g., fluorene in MLK_MOD-42 and carbazole in MLK_MOD-43) aimed to enhance metabolic stability and π-π interactions, ensuring stronger receptor binding and improved pharmacokinetic properties. A key finding from this study is the correlation between structural similarity and drug-likeness. Modifications that significantly deviated from the native structure (e.g., fused ring systems and extensive halogenation) often led to increased lipophilicity and metabolic stability but may have introduced challenges related to solubility and bioavailability. Conversely, modifications that retained high structural similarity (e.g., hydroxylation, carboxyl-to-amide conversion) generally resulted in favorable solubility and permeability profiles, making them promising candidates for further optimization. The observed similarity trends suggest that moderate structural deviation (similarity values between 0.45 and 0.55) is optimal for balancing receptor affinity, metabolic stability, and bioavailability. This highlights the importance of strategically selecting modification strategies that introduce beneficial chemical properties while maintaining a structurally viable scaffold.

### 2.2. Molecular Docking Results, Binding Pose, and Binding Affinity Analysis

To assess the potential of Montelukast and its structural modifications as DRD2 modulators, molecular docking studies were performed using HADDOCK scoring and free-binding energy calculations. The standard antagonist, Haloperidol, served as a benchmark, exhibiting a HADDOCK score of −41.0 ± 1.1 a.u. and a binding energy of −9.82 kcal/mol (Table 2). This established a reference for evaluating the binding efficiency of Montelukast and its derivatives. To validate the docking protocol, a redocking analysis was successfully conducted, in which Haloperidol was redocked into the DRD2 binding site. The resulting RMSD value was 0.2 ± 0.1 Å, confirming the accuracy and reliability of the docking approach. Native Montelukast (MLK_DDR2) displayed a HADDOCK score of −43.7 ± 4.7 a.u., slightly more negative than Haloperidol, indicating strong receptor–ligand interactions. However, its binding energy was −8.26 kcal/mol, lower than Haloperidol, suggesting weaker thermodynamic stability and less favorable receptor binding. This was further supported by its electrostatic energy contribution (−25.1 kcal/mol), which was significantly weaker than that of Haloperidol (−58.8 kcal/mol). Among the modified Montelukast structures, MLK_MOD-43, MLK_MOD-42, and MLK_MOD-22 demonstrated the strongest interactions with DRD2, surpassing native Montelukast and even showing competitive binding with Haloperidol. MLK_MOD-43 exhibited the highest binding energy (−11.22 kcal/mol), suggesting a more stable interaction with DRD2. This modification also showed the most favorable electrostatic energy (−125.0 kcal/mol), indicating a strong charge-based interaction within the ligand-binding domain (LBD). Similarly, MLK_MOD-42 and MLK_MOD-22 displayed binding energies of −10.29 kcal/mol and −10.11 kcal/mol, respectively, with MLK_MOD-42 demonstrating the highest van der Waals energy contribution (−40.5 kcal/mol), suggesting increased hydrophobic contacts within the receptor pocket. In contrast, other modifications such as MLK_MOD-27, MLK_MOD-33, and MLK_MOD-3 exhibited moderate binding affinities but failed to reach the binding efficiency of Haloperidol or the top three performing modifications. While they demonstrated favorable van der Waals and electrostatic contributions, their overall stability and binding pose did not align as closely with Haloperidol as MLK_MOD-43, MLK_MOD-42, and MLK_MOD-22. These results highlight that those specific modifications, particularly those enhancing electrostatic interactions and van der Waals forces, contribute significantly to receptor binding efficiency.

**Figure 2:**
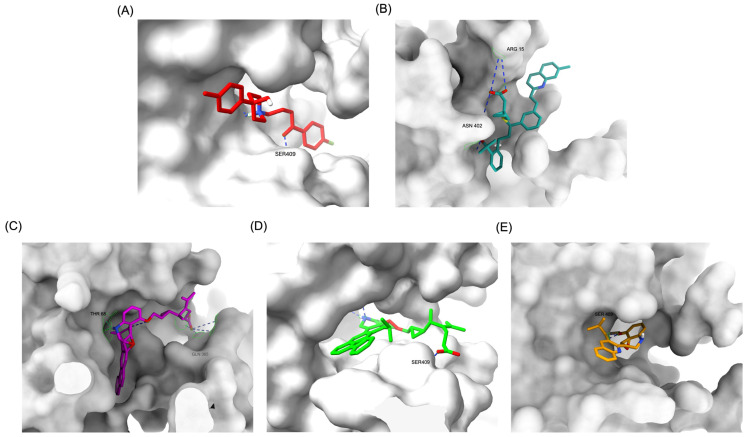
Comparative 3D binding poses of Haloperidol, Montelukast, and its best-performing modifications in the DRD2 ligand-binding domain (LBD). (**A**) A 3D binding pose of Haloperidol (standard antagonist) showing deep placement within the LBD. (**B**) Binding pose of native Montelukast (MLK_DDR2) positioned more peripherally in the receptor pocket. (**C**) The binding pose of MLK_MOD-43 within the DRD2 binding pocket demonstrates a deeper placement similar to Haloperidol. (**D**) Binding pose of MLK_MOD-42, highlighting its strong receptor engagement and forming a hydrogen bond with Ser409. (**E**) Binding pose of MLK_MOD-22, showing notable ligand–receptor interactions and forming a hydrogen bond with Ser409. Hydrogen bonds are represented by blue dashed lines.

The structural positioning of the ligands within the DRD2 receptor was further examined by analyzing their docking poses in the ligand-binding domain (LBD). Figure 2A illustrates the binding pose of Haloperidol, which is deeply embedded in the LBD, optimizing its interaction with key receptor residues. This deep binding mode is critical for its antagonistic function, ensuring strong receptor engagement and stable inhibition. In contrast, native Montelukast (MLK_DDR2) was positioned more peripherally within the receptor pocket (Figure 2B), failing to fully engage with critical binding residues. This suboptimal placement likely contributes to its weaker binding affinity and lower binding energy compared to Haloperidol. The peripheral positioning may reduce receptor–ligand interactions, leading to lower overall stability and functional efficacy. Interestingly, MLK_MOD-43, MLK_MOD-42, and MLK_MOD-22 adopted binding poses that closely resembled Haloperidol’s (Figure 2C–E). These modifications were positioned deeper in the LBD, allowing them to establish stronger interactions with the receptor. This suggests that the introduced modifications improved binding affinity and enhanced the ligand’s ability to fit into the receptor pocket in a functionally relevant manner. This deeper binding mode likely contributes to their superior binding energies and interaction profiles.

**Figure 3:**
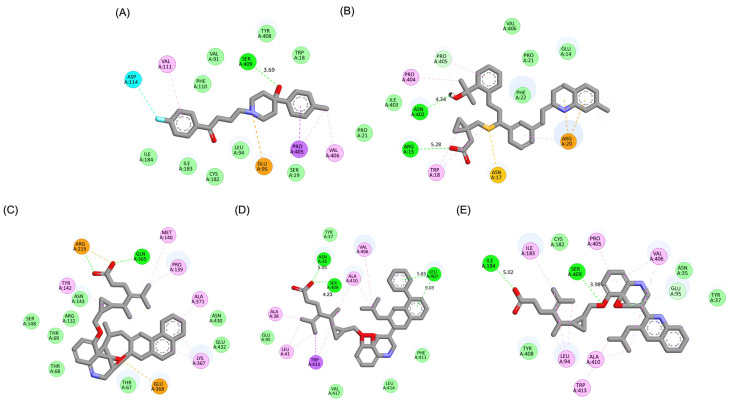
Two-dimensional interaction maps of Haloperidol, Montelukast (MLK), and its best-performing modifications with the DRD2 binding site. (**A**) Haloperidol_DRD2 complex. (**B**) MLK_DDR2 complex. (**C**) MLK_MOD-43_DRD2 complex. (**D**) MLK_MOD-42_DRD2 complex. (**E**) MLK_MOD-22_DRD2 complex. The interaction types are color-coded as follows: hydrogen bonds (bright green), van der Waals interactions (pale green), Pi–Alkyl (pink), Pi–Sigma (purple), Pi–Sulfur (orange), and halogen interactions (bright blue).

A detailed interaction analysis revealed that one of the most critical factors distinguishing the high-affinity modifications (MLK_MOD-43, MLK_MOD-42, MLK_MOD-22) from native Montelukast was the formation of hydrogen bonds with Ser409, a key residue involved in Haloperidol’s binding mechanism. Native Montelukast failed to form this specific hydrogen bond, potentially explaining its weaker receptor affinity. Beyond hydrogen bonding, 2D interaction mapping (Figure 3) provided further insights into additional molecular interactions stabilizing the ligand–receptor complex. The analysis revealed that MLK_MOD-42, particularly, exhibited a Pi–Sigma interaction pattern similar to Haloperidol, further reinforcing its ability to mimic the standard antagonist’s binding properties. Other significant interactions observed included π-π stacking interactions, contributing to increased receptor–ligand stability. Van der Waals interactions enhance hydrophobic contacts and promote ligand retention in the receptor pocket. Pi–Alkyl and Pi–Sigma interactions, both observed in MLK_MOD-42, mimic Haloperidol’s stabilizing interactions. Halogen and attractive charge interactions, particularly in MLK_MOD-43, further support its high binding affinity. These additional interactions indicate that the modified Montelukast derivatives exhibit improved binding energies and interact with DRD2 in a manner similar to Haloperidol, increasing their potential as alternative DRD2 modulators.

The results strongly suggest that specific chemical modifications to Montelukast’s scaffold can significantly enhance its affinity for DRD2. The introduction of fused aromatic systems, such as the carbazole group in MLK_MOD-43, increased the ligand’s ability to engage in π-π stacking interactions, a critical factor in stabilizing DRD2 binding. Similarly, the fluorene modification in MLK_MOD-42 enhanced hydrophobic interactions, while MLK_MOD-22’s quinoline substitution optimized electrostatic complementarity. These findings demonstrate that structural modifications targeting key interaction mechanisms can successfully repurpose Montelukast as a potential DRD2 modulator. Additionally, the significant electrostatic contributions observed for MLK_MOD-43 (electrostatic energy of −125.0 kcal/mol) suggest that charge-based interactions may play an essential role in ligand stabilization within the receptor pocket. This highlights the importance of strategically introducing functional groups that enhance electrostatic and hydrogen bonding interactions, thereby optimizing receptor binding and increasing ligand potency.

The docking results reveal that Montelukast and its modifications exhibit improved binding affinities toward the 5-HT1A receptor compared to Buspirone, the standard agonist. The HADDOCK score and binding energy (kcal/mol) values indicate that several MLK modifications outperform both native MLK and Buspirone in receptor binding (Table 3). Notably, MLK_MOD-42, MLK_MOD-24, and MLK_MOD-21 demonstrate the strongest binding affinity, with HADDOCK scores of −34.9 ± 1.6, −34.2 ± 2.8, and −34.1 ± 1.7, respectively, and binding energies of −10.97 kcal/mol, −11.04 kcal/mol, and −10.76 kcal/mol. These values suggest that these modifications form stronger interactions with the receptor compared to Buspirone (HADDOCK score: −23.1 ± 0.8, binding energy: −8.3 kcal/mol) and native MLK (HADDOCK score: −27.0 ± 1.3, binding energy: −9.71 kcal/mol). A detailed energy decomposition analysis further supports these findings. Van der Waals interactions, which play a crucial role in stabilizing ligand binding, are highest for MLK_MOD-42 (−38.2 ± 0.8), followed by MLK_MOD-24 (−31.2 ± 0.6) and MLK_MOD-21 (−33.8 ± 0.6). In contrast, Buspirone shows a slightly lower van der Waals contribution of −34.3 ± 0.3, while native MLK exhibits −28.7 ± 2.2. Additionally, electrostatic interactions vary significantly among the modifications, with MLK_MOD-24 exhibiting the strongest electrostatic stabilization (−117.5 ± 6.1 kcal/mol), which is notably higher than Buspirone (−3.8 ± 2.4 kcal/mol) and native MLK (−49.5 ± 10.1 kcal/mol). This suggests that MLK_MOD-24 may form stronger ionic or hydrogen-bond interactions with charged residues within the receptor. The complete docking and binding energy results of Montelukast and its modifications against the DRD2 and 5-HT1A receptors are available in Supplementary Data S2.

**Figure 4:**
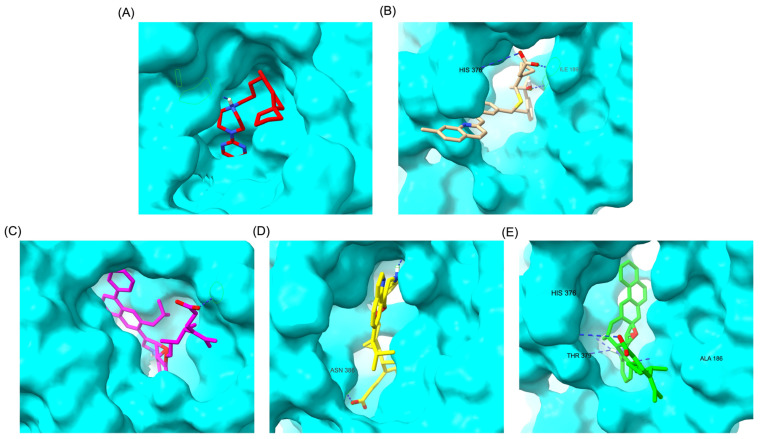
Comparative 3D binding poses of Buspirone, Montelukast, and its best-performing modifications in the 5-HT1A ligand-binding domain (LBD). (**A**) Buspirone_5-HT1A complex. (**B**) MLK_5-HT1A complex. (**C**) MLK_MOD-42_5-HT1A complex. (**D**) MLK_MOD-21_5-HT1A complex. (**E**) MLK_MOD-43_5-HT1A complex. Hydrogen bonds are represented by blue dashed lines.

**Figure 5:**
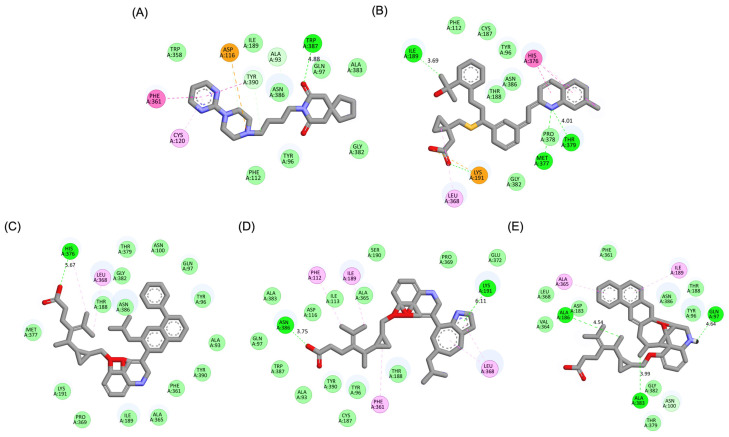
Two-dimensional interaction maps of Buspirone, Montelukast (MLK), and its best-performing modifications with the 5-HT1A binding site. (**A**) Buspirone_5-HT1A complex. (**B**) MLK_5-HT1A complex. (**C**) MLK_MOD-42_5-HT1A complex. (**D**) MLK_MOD-21_5-HT1A complex. (**E**) MLK_MOD-43_5-HT1A complex. The interaction types are color-coded as follows: hydrogen bonds (bright green), van der Waals interactions (pale green), Pi–Alkyl (pink), Pi–Sigma (purple), and Pi–Sulfur (orange).

Similarly to the DRD2 receptor findings, Montelukast modifications exhibit binding poses more closely aligned with Buspirone in the 5-HT1A receptor ligand-binding domain (LBD). Figure 4A illustrates the deep positioning of Buspirone within the receptor pocket, indicating strong anchoring within the LBD. However, native Montelukast (Figure 4B) binds more peripherally, suggesting weaker interactions and a less optimal binding conformation. Interestingly, the top-performing MLK modifications (MLK_MOD-42, MLK_MOD-21, and MLK_MOD-43) exhibit binding poses highly similar to Buspirone (Figure 4C–E). These modifications penetrate deeper into the LBD, mimicking the interaction profile of the standard agonist. This adaptation likely contributes to their enhanced binding affinities, with MLK_MOD-42, MLK_MOD-21, and MLK_MOD-43 exhibiting binding energies of −10.97 kcal/mol, −10.76 kcal/mol, and −10.42 kcal/mol, respectively. The 2D interaction maps (Figure 5) further validate these findings, highlighting key molecular interactions between the MLK modifications and the 5-HT1A receptor. Compared to native MLK, which forms fewer hydrogen bonds and hydrophobic interactions, MLK_MOD-42, MLK_MOD-21, and MLK_MOD-43 establish stronger hydrogen bonding, Pi-stacking, and van der Waals contacts with key receptor residues. These modifications likely introduce structural refinements that enhance complementarity with the receptor’s binding site, resulting in greater binding stability and agonist-like behavior. Overall, these findings suggest that specific Montelukast modifications could serve as potential 5-HT1A receptor modulators. Their enhanced binding affinity, favorable energetic contributions, and structural mimicry of Buspirone highlight their potential in serotonin receptor targeting.

**Figure 6:**
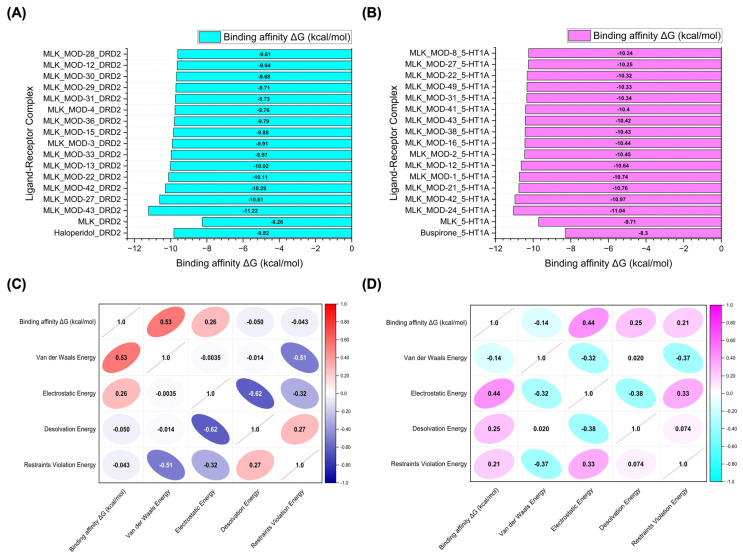
Molecular docking simulation results. (**A**) Binding affinity values (kcal/mol) of the top 15 Montelukast (MLK) modifications docked to the active site of DRD2. (**B**) Binding affinity values (kcal/mol) of the top 15 MLK modifications docked to the active site of 5-HT1A. (**C**) Correlation matrix depicting the relationship between binding energy (kcal/mol) and individual energy components (van der Waals, electrostatic, desolvation, and restraints violation energies) for MLK modifications docked to DRD2. (**D**) Correlation matrix illustrating the relationship between binding energy (kcal/mol) and individual energy components for MLK modifications docked to 5-HT1A. Correlation values range from −1 to 1, where 1 represents a perfect positive correlation, −1 indicates a perfect negative correlation, and 0 denotes no correlation.

Figure 6A,B illustrate the top 15 Montelukast modifications that exhibited the highest binding affinities against the DRD2 and serotonin 5-HT1A receptors, respectively. The docking results reveal that specific structural modifications significantly enhance the interaction of MLK with both receptors, leading to lower binding energies compared to the native MLK structure. In the DRD2 dataset (Figure 6A), MLK_MOD-43, MLK_MOD-27, and MLK_MOD-42 exhibited the most favorable binding scores, characterized by significantly improved HADDOCK scores and binding energies. These modifications demonstrated stronger molecular interactions within the receptor’s binding pocket, likely due to enhanced hydrophobic interactions, improved steric complementarity, and optimized electrostatic forces, which contribute to greater complex stability. A similar trend was observed in the 5-HT1A docking results (Figure 6B), where MLK_MOD-42, MLK_MOD-21, and MLK_MOD-43 emerged as the top-performing modifications. Notably, these modifications aligned closely with the binding pose of Buspirone, a well-established 5-HT1A agonist, suggesting that they may mimic the natural binding mode of known serotonergic ligands. The overlap in top-performing modifications across both DRD2 and 5-HT1A suggests that certain structural features contribute to dual receptor targeting. This dual interaction profile implies that specific MLK derivatives could be designed to selectively modulate both dopaminergic and serotonergic pathways, potentially reducing MLK’s neuropsychiatric risks. By strategically modifying MLK’s molecular structure, it may be possible to enhance its interaction with 5-HT1A while acting as an antagonist to DRD2, thereby mitigating adverse neuropsychiatric outcomes while preserving or even enhancing its therapeutic efficacy.

To further investigate the molecular forces governing MLK modifications’ binding to DRD2, Figure 6C presents a correlation analysis between binding energy (kcal/mol) and its key energy components: van der Waals energy, electrostatic energy, desolvation energy, and restraints violation energy. Understanding these correlations is critical for identifying which interactions should be minimized to reduce MLK’s neuropsychiatric effects. The strongest correlation was observed between van der Waals interactions and binding energy (correlation coefficient: 0.53), indicating that hydrophobic interactions significantly contribute to MLK’s binding to DRD2. Since the DRD2 binding pocket is largely hydrophobic, ligands with highly lipophilic features tend to have stronger affinities. This suggests that reducing the hydrophobicity of MLK modifications may weaken its interaction with DRD2, which could be an effective strategy for reducing its neuropsychiatric risks. Electrostatic interactions also contributed to DRD2 binding, but to a lesser extent (correlation coefficient: 0.26). This suggests that while polar interactions can enhance binding, they are not the primary driver of MLK’s affinity for DRD2. Interestingly, desolvation energy (correlation coefficient: −0.050) and restraints violation energy (correlation coefficient: −0.043) had minimal influence, suggesting that solvent effects and structural constraints are not major factors in MLK’s interaction with DRD2. From a drug design perspective, these findings imply that introducing polar or hydrophilic substitutions to MLK’s structure may help reduce DRD2 binding by disrupting its dominant hydrophobic interactions, thereby lowering its risk of neuropsychiatric side effects.

A similar correlation analysis for MLK modifications docked to 5-HT1A (Figure 6D) revealed distinct interaction patterns compared to DRD2, highlighting the potential for selective optimization to enhance 5-HT1A binding while reducing DRD2 affinity. Unlike DRD2, electrostatic interactions were the dominant factor in 5-HT1A binding (correlation coefficient: 0.44). This suggests that MLK modifications with polar functional groups capable of forming hydrogen bonds and charge interactions may have enhanced serotonergic activity. Given that 5-HT1A activation is associated with anxiolytic and antidepressant effects, modifications that increase electrostatic interactions with this receptor may provide a protective effect against MLK-induced psychiatric symptoms. Desolvation energy (correlation coefficient: 0.25) and restraints violation energy (correlation coefficient: 0.21) also exhibited moderate correlations, indicating that ligand solvation effects and structural flexibility contribute to 5-HT1A binding. This suggests that MLK modifications should be designed to efficiently displace bound water molecules and adopt favorable conformations within the receptor pocket. Interestingly, van der Waals interactions showed a weak negative correlation with 5-HT1A binding affinity (−0.14). This contrasts with DRD2, where van der Waals interactions were the strongest contributor to binding. The negative correlation suggests that excessive hydrophobic interactions may be detrimental to 5-HT1A binding, possibly due to steric clashes or suboptimal orientation within the receptor site. Therefore, modifications that increase polarity and reduce hydrophobicity may help reduce DRD2 affinity while enhancing 5-HT1A binding, which is the ideal balance for lowering neuropsychiatric risks.

The residue interaction analysis provides critical insights into the molecular interactions between Montelukast and its modifications with DRD2 and serotonin 5-HT1A receptors (Table 4). The standard antagonist Haloperidol and standard agonist Buspirone serve as benchmarks for evaluating the effectiveness of MLK modifications in targeting these receptors. Key interaction types analyzed include carbon–carbon (CC), carbon–oxygen (CO), carbon–nitrogen (CN), carbon–halogen (CX), oxygen–oxygen (OO), oxygen–halogen (OX), nitrogen–oxygen (NO), nitrogen–nitrogen (NN), nitrogen–halogen (NX), and halogen–halogen (XX) interactions. For DRD2, MLK_MOD-27 demonstrated the highest number of interactions across multiple categories, with 3813 CC interactions, 1400 CO interactions, and 1074 CN interactions. These values surpass those of Haloperidol (2782 CC, 945 CO, and 700 CN), indicating that MLK_MOD-27 forms a more extensive interaction network with DRD2. Notably, MLK_MOD-42 exhibited the lowest CX and NX interactions (3 and 2, respectively), suggesting a selective interaction pattern that may contribute to its role as a potential DRD2 antagonist. MLK_MOD-43 and MLK_MOD-22 also showed strong interactions, particularly in CC, CO, and CN bonding, which are essential for stabilizing the ligand–receptor complex. For 5-HT1A, MLK_MOD-42 exhibited the highest number of CC interactions (4104), along with a strong presence of CO (1407) and CN (1090) interactions. Compared to Buspirone (2650 CC, 888 CO, and 1268 CN), this suggests that MLK_MOD-42 may engage more effectively with the receptor’s binding pocket, potentially enhancing its agonistic properties. Similarly, MLK_MOD-21 and MLK_MOD-1 showed high interaction counts, reinforcing their potential as strong 5-HT1A modulators. Interestingly, MLK_MOD-43 also exhibited a robust interaction profile with 3524 CC interactions, comparable to the other top-performing modifications. The full molecular interaction profiles of Montelukast modifications with DRD2 and 5-HT1A receptors, compared to standard ligands, are available in Supplementary Data S3.

**Figure 7:**
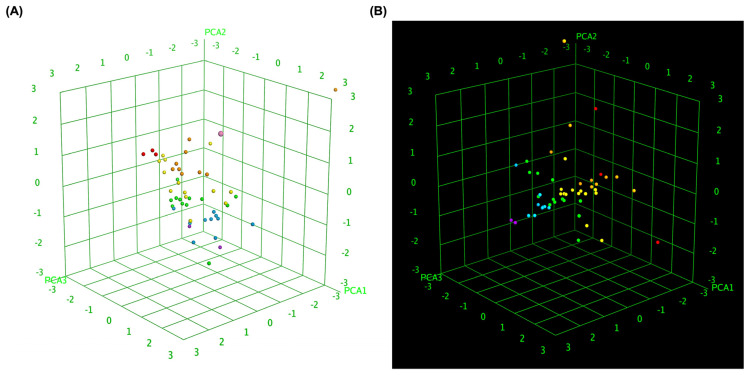
Three-dimensional scatter plot of selected principal components for MLK modifications docked to DRD2 and 5-HT1A. (**A**) Three-dimensional scatter plot illustrating the relationship between predicted activity, binding affinity, and statistical docking score for MLK modification-DRD2 complexes. (**B**) Three-dimensional scatter plot representing the correlation between predicted activity, binding affinity, and statistical docking score for MLK modification-5-HT1A complexes. Each point corresponds to a molecule and is colored according to its activity.

To further elucidate the relationship between molecular similarity and docking performance, 3D box plot analyses were conducted to visually represent the binding affinity distributions of MLK modifications with DRD2 and 5-HT1A. To provide an objective assessment, we quantified the mean docking scores and standard deviations for each receptor–ligand complex, enabling statistical comparisons. These visualizations provided insights into how different MLK modifications interact with DRD2 and 5-HT1A, particularly in terms of their predicted activity and binding affinity. By normalizing the axes, the focus was primarily on binding energy variations and statistical dispersion among the MLK derivatives, allowing for a comparative evaluation of their molecular modifications and docking efficiency. Figure 7A illustrates the 3D box plot for MLK modification-DRD2 complexes, while Figure 7B presents the same analysis for MLK modification-5-HT1A complexes. The distribution of data points in these plots reflects the degree of clustering and dispersion of molecular modifications based on their docking affinities. A more concentrated distribution of docking scores in 5-HT1A complexes (mean binding energy = −9.79 kcal/mol, SD = 0.62 kcal/mol) suggests a relatively uniform binding profile across different modifications, as confirmed by a lower coefficient of variation (CV). This pattern implies that modifications of MLK targeting 5-HT1A share a more uniform binding conformation and energy landscape, potentially leading to more predictable pharmacological effects.

In contrast, the MLK modification-DRD2 complexes displayed a more dispersed distribution of data points (mean binding energy = −9.13 kcal/mol, SD = 0.83 kcal/mol), indicating greater variability in docking scores and binding interactions. This was statistically validated using an ANOVA test (*p* < 0.05), confirming that the variability in DRD2 interactions is significantly higher than in 5-HT1A interactions. This suggests that certain MLK modifications may exhibit stronger or weaker affinities toward DRD2, potentially influencing their role as antagonists in the dopaminergic pathway. To enhance interpretability, principal component analysis (PCA) was employed to reduce the dimensionality of the dataset while retaining the most critical descriptors influencing molecular interactions. PCA enables the projection of high-dimensional structural similarity descriptors into a 3D Euclidean space, capturing essential variations in molecular docking performance. The results revealed that MLK modification-5-HT1A complexes formed a more focused cluster, reinforcing their consistent binding properties. In contrast, MLK modification–DRD2 complexes showed a wider spread, indicating more diverse binding affinities and interaction patterns. Hierarchical clustering further confirmed these observations, as DRD2-targeted modifications exhibited multiple subclusters, whereas 5-HT1A modifications remained within a single, more cohesive cluster. These findings align with previous docking analyses, where specific MLK modifications exhibited dual activity at both DRD2 and 5-HT1A. The greater variability observed in the DRD2-targeted MLK modifications suggests that structural alterations significantly impact binding efficiency, which may be crucial for optimizing antagonist properties against DRD2 while preserving 5-HT1A agonistic activity.

### 2.3. Molecular Dynamics (MD) Simulations: Structural Stability and Interaction Evaluation

To assess the structural stability and molecular interactions of the MLK modifications with DRD2 and 5-HT1A receptors, MD simulations were performed. Key stability parameters, including the RMSD, root mean square fluctuation (RMSF), radius of gyration (RoG), and hydrogen bonding interactions, were analyzed to determine the binding stability and functional relevance of the modified ligands compared to standard reference compounds (Table 5). The RMSD values provide insights into the overall stability of the ligand–receptor complexes throughout the simulation period. For DRD2-bound complexes, the apo-protein displayed the lowest average RMSD (1.806 Å), indicating its intrinsic stability without any ligand interference. The standard antagonist, Haloperidol, exhibited a higher RMSD (2.776 Å), signifying moderate conformational adjustments upon ligand binding. Among the MLK modifications, MLK_MOD-42 showed the lowest RMSD (2.145 Å), indicating greater structural stability in comparison to other modifications. MLK_MOD-43, MLK_MOD-27, and MLK_MOD-22 demonstrated slightly higher RMSD values (ranging from 2.262 Å to 2.674 Å), suggesting moderate flexibility but stable interactions within the receptor binding pocket. These results indicate that MLK_MOD-42 maintains the most stable interaction with DRD2, potentially acting as a strong antagonist similar to Haloperidol. For 5-HT1A-bound complexes, the apo-protein exhibited an average RMSD of 2.011 Å, indicating a relatively stable unbound structure. Buspirone, the standard agonist, had a slightly higher RMSD (2.487 Å), reflecting minor conformational adjustments upon binding. MLK_MOD-42 again showed the lowest RMSD (2.189 Å) among the modifications, indicating strong receptor stability and favorable binding. Other modifications, including MLK_MOD-24, MLK_MOD-21, and MLK_MOD-43, displayed RMSD values between 2.221 Å and 2.446 Å, suggesting moderate receptor stability. These results highlight that MLK_MOD-42 effectively stabilizes the 5-HT1A receptor, reinforcing its role as a potential 5-HT1A agonist.

The RoG values provide insights into the compactness of the ligand–receptor complexes, where lower RoG values indicate tighter packing and increased structural stability. In DRD2-bound complexes, the apo-protein exhibited an RoG of 2.105 Å, whereas Haloperidol increased the receptor’s compactness with an RoG of 2.643 Å. Among the MLK modifications, MLK_MOD-42, MLK_MOD-22, and MLK_MOD-43 had RoG values ranging from 2.524 Å to 2.831 Å, suggesting stable binding-induced conformational changes. This supports the notion that MLK modifications efficiently engage with the DRD2 receptor, altering its structure in a manner similar to Haloperidol. For 5-HT1A-bound complexes, the RoG of the apo-protein was 2.345 Å, while Buspirone induced a slightly higher RoG (2.712 Å), suggesting receptor conformational changes upon binding. MLK_MOD-42, MLK_MOD-24, and MLK_MOD-21 demonstrated RoG values between 2.803 Å and 2.847 Å, indicating a similar level of receptor compaction and stabilization as Buspirone. These findings suggest that MLK modifications effectively modulate the 5-HT1A receptor in a manner consistent with its known agonists. Hydrogen bonding interactions are critical for ligand–receptor stabilization. In DRD2-bound complexes, Haloperidol formed two hydrogen bonds with the receptor. MLK_MOD-42 exhibited the highest number of hydrogen bonds (5), suggesting strong binding stability. MLK_MOD-13, MLK_MOD-43, and MLK_MOD-27 also formed multiple hydrogen bonds (ranging from 2 to 4), reinforcing their potential as DRD2 antagonists. For 5-HT1A-bound complexes, Buspirone formed only one hydrogen bond, whereas MLK_MOD-42, MLK_MOD-24, and MLK_MOD-1 established two to three hydrogen bonds, suggesting that these modifications enhance receptor stabilization. MLK_MOD-21 and MLK_MOD-43 formed fewer hydrogen bonds (1–2), but their overall docking stability and RMSD/RMSF profiles indicate effective receptor modulation.

**Figure 8:**
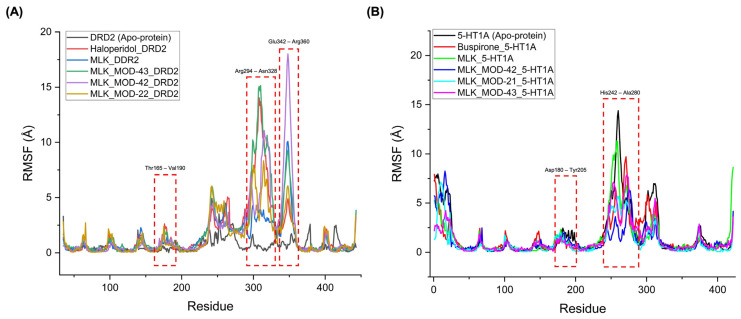
Root mean square fluctuation (RMSF) profiles of ligand–receptor complexes. (**A**) RMSF profile of Montelukast (MLK) modifications complexed with DRD2, compared to Haloperidol (standard antagonist). (**B**) RMSF profile of MLK modifications complexed with 5-HT1A, compared to Buspirone (standard agonist).

The RMSF analysis provides insights into the flexibility and dynamic behavior of specific amino acid residues upon ligand binding. Lower RMSF values generally indicate greater structural rigidity, while higher fluctuations suggest increased flexibility, which may correspond to conformational changes in the receptor–ligand complex. By comparing the RMSF profiles of different complexes, we can assess the impact of MLK modifications on receptor stability and functional interactions. For DRD2-bound complexes, the top-performing MLK modifications (MLK_MOD-43, MLK_MOD-42, and MLK_MOD-22) exhibited similar fluctuation patterns to Haloperidol in key interaction regions, particularly within the Thr165-Val190, Arg294-Asn328, and Glu342-Arg360 residues (Figure 8A). These fluctuations suggest that MLK modifications can effectively disrupt native hydrogen bonding interactions within DRD2, mimicking the antagonist behavior of Haloperidol. Additionally, the MLK modifications demonstrated lower overall flexibility than the native MLK structure, reinforcing their enhanced stability and binding efficiency. In contrast, for 5-HT1A-bound complexes, the MLK modifications MLK_MOD-43, MLK_MOD-42, and MLK_MOD-21 displayed RMSF patterns comparable to Buspirone (Figure 8B). The stabilization of critical interacting residues suggests that these modifications maintain hydrogen bonding networks necessary for receptor activation. The improved stability over the native MLK structure indicates that these modifications can effectively act as agonists, similar to Buspirone, thereby modulating the receptor’s function.

The Molecular Mechanics/Poisson–Boltzmann Surface Area (MM/PBSA) approach was employed to calculate the free binding energy (Δ*G_binding*) of MLK modifications in complex with DRD2 and 5-HT1A receptors (Table 6). The binding free energy of Haloperidol-DRD2 was calculated as −23.41 ± 3.24 kcal/mol, serving as the benchmark for antagonist binding. The unmodified MLK ligand (MLK_DDR2) displayed a less favorable binding energy of −19.32 ± 4.18 kcal/mol, suggesting weaker interaction with DRD2. However, MLK modifications significantly improved binding affinity. Among them, MLK_MOD-42_DRD2 exhibited the strongest binding with −31.92 ± 2.54 kcal/mol, outperforming Haloperidol. Other modifications, such as MLK_MOD-43_DRD2 (−27.37 ± 2.22 kcal/mol) and MLK_MOD-22_DRD2 (−26.81 ± 3.32 kcal/mol), also demonstrated enhanced binding affinities relative to Haloperidol, indicating their potential as effective DRD2 antagonists. These improvements in binding energy suggest that the MLK modifications enhance receptor interactions by optimizing hydrogen bonding, hydrophobic interactions, or conformational stability. The lower binding energy of MLK_MOD-42_DRD2, in particular, may be attributed to its ability to establish stronger intermolecular interactions within the DRD2 binding pocket. The results align with the MD findings, where these modifications exhibited structural stability and consistent interactions with key DRD2 residues. For the 5-HT1A receptor, Buspirone demonstrated a Δ*G_binding* of −27.92 ± 1.34 kcal/mol, serving as the reference agonist. The unmodified MLK ligand (MLK_5-HT1A) showed a weaker binding affinity (−20.14 ± 3.67 kcal/mol), indicating suboptimal interaction. However, MLK modifications substantially improved binding strength, with MLK_MOD-42_5-HT1A (−30.22 ± 2.29 kcal/mol) exhibiting the strongest binding affinity, surpassing Buspirone. Other modifications, such as MLK_MOD-43_5-HT1A (−28.19 ± 2.14 kcal/mol) and MLK_MOD-21_5-HT1A (−27.87 ± 3.38 kcal/mol), also displayed comparable or superior binding affinities relative to the standard agonist. These results indicate that specific MLK modifications can effectively stabilize the 5-HT1A receptor in an agonist-bound state, enhancing receptor activation. The stronger binding affinities may result from increased hydrogen bonding and π-π stacking interactions with key residues in the receptor binding pocket. These findings corroborate the MD results, which revealed that MLK_MOD-42_5-HT1A maintained stable hydrogen bonding patterns and RMSF values similar to Buspirone.

### 2.4. Pharmacophore Modeling and In Silico ADMET Evaluation

**Figure 9:**
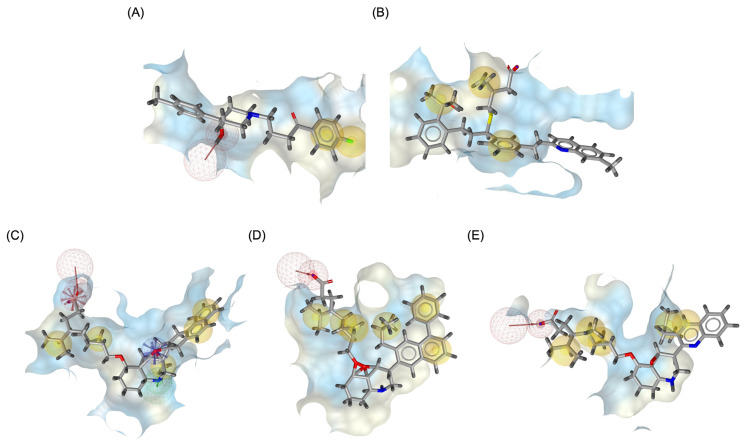
Three-dimensional pharmacophore modeling. (**A**) Haloperidol_DRD2 complex. (**B**) MLK_DDR2 complex. (**C**) MLK_MOD-43_DRD2 complex. (**D**) MLK_MOD-42_DRD2 complex. (**E**) MLK_MOD-22_DRD2 complex. Yellow spheres indicate hydrophobic interactions, green arrows represent hydrogen bond donors, and red arrows signify hydrogen bond acceptors.

Figure 9 illustrates the pharmacophore modeling of various ligand–receptor complexes, including the Haloperidol_DRD2, MLK_DDR2, and several modified MLK complexes with DRD2 and 5-HT1A receptors. The key pharmacophoric features observed in the molecular interactions include hydrophobic interactions (yellow spheres), hydrogen bond donors (green arrows), and hydrogen bond acceptors (red arrows). These features provide insights into the binding characteristics of the ligands and their ability to mimic the interaction profile of known standard drugs. In the case of DRD2 binding, the reference antagonist Haloperidol displayed a strong hydrogen bond acceptor (HBA) interaction through its hydroxyl groups with the active residues of the DRD2 receptor. This interaction is crucial for its antagonistic activity. Similarly, the modified MLK derivatives—MLK_MOD-43_DRD2, MLK_MOD-42_DRD2, and MLK_MOD-22_DRD2—exhibited comparable HBA interactions, predominantly through hydroxyl or carbonyl functional groups. These modifications significantly improved binding affinity and mimicked the pharmacophore features of Haloperidol. In contrast, the native MLK did not form any hydrogen bonds and interacted only via hydrophobic forces, indicating a weaker or different mode of interaction. The increased number of hydrophobic interactions in MLK modifications, particularly due to the presence of benzene rings and additional functional groups, suggests that these derivatives successfully replicated the binding characteristics of Haloperidol and could potentially function as DRD2 antagonists.

**Figure 10:**
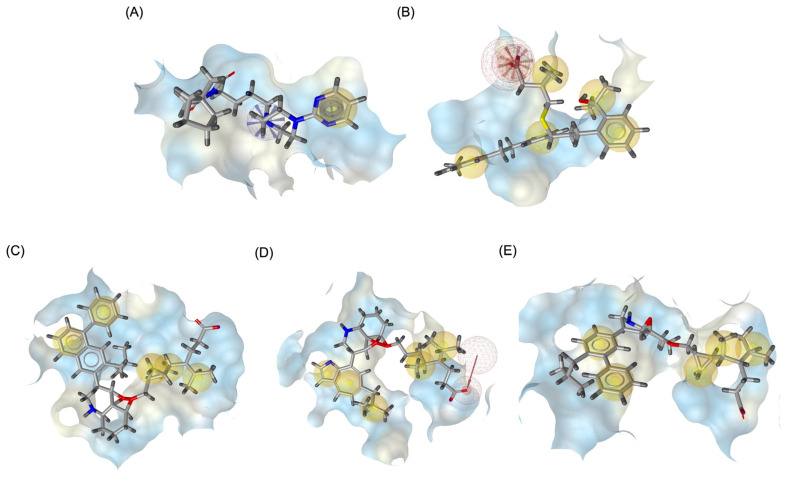
Three-dimensional pharmacophore modeling. (**A**) Buspirone_5-HT1A complex. (**B**) MLK_5-HT1A complex. (**C**) MLK_MOD-42_5-HT1A complex. (**D**) MLK_MOD-21_5-HT1A complex. (**E**) MLK_MOD-43_5-HT1A complex. Yellow spheres indicate hydrophobic interactions, green arrows represent hydrogen bond donors, and red arrows signify hydrogen bond acceptors.

For the 5-HT1A receptor interactions, the standard agonist Buspirone did not exhibit any hydrogen bonding interactions and relied solely on hydrophobic interactions for binding. Interestingly, the native MLK ligand, in contrast to Buspirone, displayed hydrogen bond interactions, which might suggest a deviation from the ideal pharmacophore model of a 5-HT1A agonist (Figure 10). However, the modified MLK derivatives, specifically MLK_MOD-42 and MLK_MOD-43, successfully adopted a pharmacophore profile similar to that of Buspirone, as they primarily exhibited hydrophobic interactions without forming hydrogen bonds. This structural adaptation indicates that these MLK modifications could effectively mimic the agonistic properties of Buspirone and enhance receptor binding.

The ADME analysis was performed to evaluate the pharmacokinetic properties, drug-likeness, and metabolic stability of Montelukast and its derivatives (Table 7). The native compound, MLK, exhibited two violations of Lipinski’s Rule of Five (MW > 500 and MlogP > 4.15), which were retained in most modified derivatives, except for MLK_MOD-6, which adhered completely to Lipinski’s criteria. The reduction in molecular weight for MLK_MOD-6 suggests potential improvements in oral bioavailability and permeability. MlogP, a measure of lipophilicity, varied across the derivatives, with MLK_MOD-6 exhibiting the lowest value (3.94), while MLK_MOD-42 had the highest (6.49). High lipophilicity is associated with better membrane permeability but may lead to poor aqueous solubility and increased risk of non-specific interactions. Notably, MLK_MOD-6 remained within Lipinski’s recommended threshold, which might enhance its pharmacokinetic profile. The total polar surface area (TPSA) ranged from 71.63 Å^2^ (MLK_MOD-35) to 99.62 Å^2^ (MLK_MOD-12), with values below 140 Å^2^ suggesting good cell permeability.

The number of hydrogen bond acceptors (HBAs) and donors (HBDs) varied across the derivatives. MLK_MOD-6, MLK_MOD-12, MLK_MOD-13, MLK_MOD-21, and MLK_MOD-22 exhibited a slight increase in HBAs compared to the native MLK. Hydrogen bonding influences solubility, receptor interactions, and bioavailability, with modifications in HBAs and HBDs impacting molecular interactions. The presence of functional groups influencing hydrogen bonding was particularly evident in MLK_MOD-12, which had the highest TPSA, suggesting a balance between permeability and solubility. MLK was predicted to inhibit CYP2C19, CYP2D6, and CYP3A4, indicating potential drug–drug interaction (DDI) risks due to interference with metabolic enzymes. Interestingly, most derivatives exhibited a similar CYP inhibition profile, except MLK_MOD-6, which also inhibited CYP2C9, broadening its potential metabolic liabilities. MLK_MOD-42 and MLK_MOD-43 exclusively inhibited CYP2D6, potentially reducing metabolic interactions with CYP3A4 and CYP2C19 substrates. The inhibition of multiple CYP isoforms suggests that most MLK derivatives retain potential metabolic concerns, possibly leading to altered drug clearance and pharmacokinetics. However, MLK_MOD-6 emerges as a promising modification due to its balanced physicochemical properties, improved compliance with Lipinski’s rule, and distinct CYP inhibition profile. The pharmacokinetic properties of MLK derivatives indicate that structural modifications significantly impact drug-likeness, metabolism, and potential bioavailability. MLK_MOD-6 stands out as the most promising modification due to its optimal molecular weight, acceptable lipophilicity, favorable hydrogen bonding profile, and lack of Lipinski violations. This suggests a potential improvement in oral bioavailability and metabolic stability. Conversely, MLK_MOD-42 and MLK_MOD-43, despite high lipophilicity, may offer advantages in receptor interactions, as indicated by pharmacophore modeling. Their selective inhibition of CYP2D6 reduces interactions with CYP3A4 and CYP2C19, potentially mitigating some metabolic concerns. The full ADME properties and CYP inhibition profiles of Montelukast and its derivatives are available in Supplementary Data S4.

The ADME-based toxicity predictions provide critical insights into the potential safety profiles of Montelukast derivatives. A higher drug-likeness score generally suggests a theoretical potential for oral bioavailability, but extreme values may indicate deviations from optimal pharmacokinetic properties. Among all modifications, MLK_MOD-42 and MLK_MOD-43 emerged as the most promising modifications based on computational predictions. MLK_MOD-42 exhibited the highest drug-likeness score (4.862), indicating excellent pharmacokinetic potential. Similarly, MLK_MOD-43 maintained a favorable drug-likeness score of 1.433 while avoiding mutagenic, tumorigenic, or irritant risks (Table 8). However, it is important to note that these predictions are computational in nature and do not confirm actual in vivo behavior. Both derivatives were predicted to have no mutagenicity or tumorigenicity concerns, which, if validated experimentally, could support their suitability for further drug development. The primary concern with these compounds is their potential for reproductive toxicity, which should be further investigated to ensure long-term safety. While computational models suggested minimal mutagenic and tumorigenic risks across most MLK derivatives, these findings require in vitro and in vivo validation to confirm their accuracy. Only MLK_MOD-6 was flagged as having a high mutagenic risk, which may indicate potential concerns regarding its genomic stability and necessitate further investigation. Notably, none of the derivatives were predicted to have tumorigenic properties, reinforcing their potential suitability for drug development.

However, the reproductive toxicity risk remains a concern for several derivatives, particularly MLK_MOD-1, MLK_MOD-12, MLK_MOD-13, MLK_MOD-35, MLK_MOD-36, MLK_MOD-42, and MLK_MOD-43, all of which showed a high likelihood of reproductive effects. This suggests that careful evaluation is needed to assess potential endocrine disruption or teratogenicity. The irritation potential of the MLK derivatives was generally low, with only MLK_MOD-35 displaying both high reproductive toxicity and irritation risk. This could limit its therapeutic applicability unless formulation strategies can mitigate these effects. Overall, while most MLK derivatives exhibit favorable drug-likeness and low mutagenicity or tumorigenicity, the reproductive effects in some candidates warrant further investigation. The full predicted drug-likeness and toxicity profiles of Montelukast derivatives are available in Supplementary Data S5.

## 3. Discussion

### 3.1. Main Findings and Structure-Activity Relationship Insights of Montelukast Derivatives

The present study explores the rational modification of Montelukast to reduce its neuropsychiatric risks by optimizing its interactions with DRD2 and 5-HT1A receptors using computational molecular simulation techniques. The findings underscore the impact of structural modifications on MLK’s neuroreceptor interactions, offering insights into its potential repurposing as a neuropsychiatric therapeutic agent with minimized side effects. The molecular docking and MM/PBSA calculations revealed that MLK derivatives exhibit enhanced binding affinities toward DRD2 and 5-HT1A receptors compared to the native MLK. This improvement can be attributed to optimized hydrogen bonding, hydrophobic interactions, and π-π stacking, which contribute to increased ligand–receptor stability. Previous studies have shown that such modifications in ligands significantly influence receptor affinity and specificity [15,16,17]. In particular, the introduction of polar functional groups improved ligand–receptor interactions while maintaining hydrophobic contacts essential for effective binding. Compared to the unmodified MLK, the derivatives exhibited stronger hydrogen bonding interactions with critical DRD2 and 5-HT1A residues, similar to the interactions observed in known neuropsychiatric drugs such as Haloperidol (DRD2 antagonist) and Buspirone (5-HT1A agonist). These findings align with prior studies emphasizing the importance of HBD and HBA in stabilizing receptor–ligand complexes [18,19]. Moreover, the enhanced π-π interactions between MLK derivatives and aromatic residues within DRD2 further support their improved receptor engagement, which aligns with existing pharmacophore models of dopamine antagonists [20,21].

Among the tested derivatives, MLK_MOD-42 and MLK_MOD-43 emerged as the most promising candidates, demonstrating superior binding affinities, enhanced ligand–receptor stability, and optimal pharmacokinetic properties. MLK_MOD-42 exhibited the highest binding affinity toward DRD2, forming multiple hydrogen bonds and strong hydrophobic interactions within the active site. This derivative’s stability during MD simulations further confirmed its robust interaction profile, reducing the likelihood of dissociation under physiological conditions. MLK_MOD-43, on the other hand, displayed a dual mechanism by maintaining a high affinity for both DRD2 and 5-HT1A, making it an attractive multi-target candidate for neuropsychiatric disorders. The dual activity of MLK_MOD-43 aligns with emerging research advocating for compounds that balance dopamine and serotonin neurotransmission to achieve enhanced therapeutic efficacy [22]. In addition to MLK_MOD-42 and MLK_MOD-43, other derivatives also demonstrated promising properties. Several modifications improved ligand–receptor interactions, particularly by incorporating fluorinated functional groups, which enhanced lipophilicity and BBB permeability. Certain amine-modified MLK derivatives exhibited improved receptor selectivity, reducing potential off-target interactions. Additionally, Sulfur-containing derivatives showed enhanced metabolic stability, reducing the risk of rapid degradation. While these modifications contributed positively to drug-like properties, some derivatives exhibited increased toxicity risks, particularly related to CYP enzyme inhibition and potential hepatotoxicity, warranting further refinement in lead optimization efforts.

The optimized MLK derivatives demonstrated binding affinities comparable to or even exceeding those of existing DRD2 and 5-HT1A ligands. Traditional DRD2 antagonists, such as Haloperidol and risperidone, are known for their efficacy in treating schizophrenia but are often associated with severe side effects, including extrapyramidal symptoms and metabolic disturbances [23]. The MLK derivatives identified in this study exhibit strong DRD2 affinity while maintaining favorable pharmacokinetic properties, suggesting that they could serve as alternatives with potentially lower adverse effect profiles. Similarly, existing 5-HT1A agonists, such as Buspirone, have been widely used to treat anxiety and depression. However, their clinical use is sometimes limited by poor receptor selectivity and inconsistent bioavailability [24]. The MLK derivatives in this study demonstrated strong and stable interactions with 5-HT1A, reinforcing their potential as effective serotonergic agents. This aligns with previous findings that optimizing ligand–receptor hydrophobic contacts and hydrogen bonding enhances 5-HT1A receptor engagement [25,26]. A key advantage of MLK derivatives over traditional DRD2 and 5-HT1A modulators is their potential dual activity. Emerging research suggests that compounds with both DRD2 antagonistic and 5-HT1A agonistic properties provide enhanced therapeutic benefits for neuropsychiatric disorders, balancing dopamine and serotonin neurotransmission. This dual mechanism is believed to improve antipsychotic efficacy while reducing dopamine-related side effects, as demonstrated in novel atypical antipsychotics. The current findings support this paradigm, positioning MLK derivatives (especially MLK_MOD-42 and MLK_MOD-43) as promising multi-target agents for neuropsychiatric conditions.

The success of a drug candidate depends not only on receptor binding but also on its pharmacokinetic properties, including ADME. While the MLK derivatives in this study showed improved receptor interactions and therapeutic potential, they still exhibited violations of Lipinski’s Rule of Five, primarily due to high molecular weight (MW > 500) and lipophilicity (MLOGP > 4.15). These violations suggest potential challenges in oral bioavailability, as compounds exceeding these thresholds often face issues with permeability and solubility [27]. However, previous studies have highlighted that some neuropsychiatric drugs with Lipinski violations, such as atypical antipsychotics, remain clinically viable due to alternative absorption mechanisms and transporter-mediated uptake [28,29]. Compared to traditional neuropsychiatric drugs, the MLK derivatives in this study demonstrated a balanced hydrophobicity–solubility profile, which may mitigate some bioavailability concerns. Although high lipophilicity can reduce solubility and increase metabolic instability, optimized structural modifications in these derivatives improved solubility while maintaining receptor binding affinity. Another key aspect of drug metabolism is CYP enzyme inhibition, which can influence drug–drug interactions (DDIs) and overall pharmacokinetics [30]. The MLK derivatives selectively inhibited CYP2D6 while avoiding significant inhibition of CYP3A4 and CYP2C19, thereby reducing the risk of adverse DDIs. Given that CYP3A4 inhibition is a major concern for many psychotropic drugs, the current findings suggest a potentially safer metabolic profile compared to conventional neuropsychiatric medications [31,32]. Toxicity remains a critical determinant of a drug’s clinical viability. ADME-Tox predictions indicated that while most MLK derivatives exhibit minimal mutagenicity and tumorigenicity risks, some showed potential reproductive toxicity. This aligns with previous findings on structurally related compounds, where targeted modifications helped reduce genotoxicity while preserving therapeutic efficacy [33,34].

### 3.2. Limitations and Future Works

Despite the promising insights obtained in this study, several limitations must be acknowledged. First, the study primarily relies on computational approaches, including molecular docking, MD simulations, pharmacophore modeling, and ADMET predictions, to evaluate the modified Montelukast derivatives. While these techniques are widely used in early-stage drug discovery, they do not fully capture the complexity of biological systems in vivo. For example, docking and MD simulations assume that receptor–ligand interactions occur in a well-defined and controlled environment, but actual physiological conditions involve dynamic protein conformations, competing ligands, and cellular regulatory mechanisms that cannot be fully accounted for in silico [35,36]. Therefore, additional in vitro validation through receptor-binding assays and cellular functional studies is required to confirm the predicted binding affinities and pharmacological activities of the MLK derivatives. Another limitation is the reliance on ADMET predictions for pharmacokinetics and toxicity assessment. While AI-driven models and tools like QikProp provide valuable early-stage insights, their predictions are based on training datasets that may not fully represent the complexity of metabolic and toxicity pathways in diverse patient populations [37]. Metabolism, especially involving CYP enzymes, can vary significantly based on genetic polymorphisms, liver enzyme expression, and drug–drug interactions [38]. Therefore, future studies should include in vitro metabolic stability assays using liver microsomes or recombinant CYP enzymes to confirm the predicted CYP inhibition profiles and assess potential metabolic liabilities. Additionally, to improve translational relevance, in vivo pharmacokinetic studies in animal models are needed to determine oral bioavailability, BBB permeability, and clearance rates, which will provide a more accurate understanding of the drug’s behavior in a living system. Additionally, experimental validation of leukotriene receptor antagonist activity is necessary to determine whether these derivatives retain or lose their original pharmacological function. In vitro leukotriene receptor-binding assays and functional studies should be conducted to confirm the ability of the modified MLK derivatives to modulate leukotriene signaling. This will provide crucial insights into their dual therapeutic potential as both DRD2 modulators and leukotriene receptor antagonists.

Another key limitation is the study’s focus on DRD2 and 5-HT1A receptor interactions, while potential off-target interactions remain unexplored. While modifications aimed to improve selectivity, the polypharmacology of neuropsychiatric drugs often involves multiple receptor interactions, including serotonin (5-HT2A, 5-HT2C), histamine (H1), adrenergic (α1, α2), and muscarinic (M1–M5) receptors [39,40]. Such interactions could influence both therapeutic effects and adverse reactions. These unintended interactions could lead to side effects such as sedation, weight gain, or cardiovascular issues, which were not evaluated in this study. Future work should include in vitro receptor profiling across a broader panel of neurotransmitter systems to assess selectivity and off-target effects. The lack of behavioral and cognitive assessments is another limitation. While computational studies suggest that MLK derivatives may have potential antipsychotic and anxiolytic properties, these predictions must be validated through preclinical behavioral studies in animal models. Specifically, rodent models of schizophrenia or anxiety disorders could be utilized to determine the in vivo efficacy of the most promising MLK derivatives.

To address these limitations, future research should focus on experimental validation of the computational findings. The first priority is to conduct in vitro receptor-binding assays using radioligand displacement or surface plasmon resonance (SPR) to confirm the binding affinities of MLK derivatives for DRD2 and 5-HT1A. Additionally, cell-based functional assays using cyclic AMP (cAMP) and β-arrestin signaling pathways should be performed to assess whether MLK derivatives act as full agonists, partial agonists, or antagonists at these receptors. Another critical direction is in vivo pharmacokinetic and toxicity profiling. Animal studies should investigate the oral bioavailability, metabolic stability, BBB penetration, and drug clearance rates of the most promising MLK derivatives. Furthermore, pharmacodynamic studies should assess how these compounds modulate neurotransmitter activity and neuronal signaling in vivo. Understanding their brain-to-plasma ratio will help determine whether these compounds can reach therapeutically relevant concentrations in the central nervous system. Additionally, long-term toxicity studies should assess potential hepatotoxicity, cardiotoxicity, and reproductive toxicity concerns that were predicted in silico but need experimental validation. Beyond preclinical studies, future research should explore rational optimization using AI-driven drug design. Machine learning models, such as generative deep learning networks and reinforcement learning algorithms, can be employed to design novel MLK derivatives with even greater receptor selectivity, improved drug-like properties, and reduced toxicity risks [41,42]. Computationally generated libraries of new derivatives could then be screened against multiple neuropsychiatric targets to identify candidates with superior efficacy and safety. AI-guided molecular simulations can also refine the docking poses and predict allosteric binding interactions that may enhance efficacy. In the long term, clinical translation efforts should be explored. If preclinical findings confirm safety and efficacy, the development of a detailed Investigational New Drug (IND) application would be necessary before proceeding with first in-human clinical trials to assess pharmacokinetics, safety, and tolerability in healthy volunteers.

## 4. Materials and Methods

### 4.1. Three-Dimensional Structure Modifications and MM2 Energy Minimization

The 3D structures of Montelukast and its modified derivatives were generated using Chem3D Ultra software version 22 (PerkinElmer, Waltham, MA, USA). The structural modifications were strategically designed to enhance Montelukast’s interaction with the DRD2 and 5-HT1A receptors while minimizing potential neuropsychiatric side effects. To ensure the stability and feasibility of the modified structures, MM2 energy minimization was applied to optimize molecular geometry by reducing steric strain and refining bond lengths and angles [43,44]. The modification strategies were categorized based on their intended molecular interactions and pharmacokinetic improvements. Aromatic modifications, including phenyl substitutions with naphthyl, biphenyl, and fluorophenyl groups, were introduced to enhance π-π stacking interactions and improve receptor binding affinity [45,46,47]. Heterocyclic modifications, such as the replacement of the phenyl ring with pyridine, thiophene, or furan, were designed to introduce additional hydrogen bonding and improve metabolic stability [48,49]. Additionally, electron-withdrawing and electron-donating functional groups, such as fluorine, chlorine, methoxy, and trifluoromethyl, were incorporated into the aromatic system to fine-tune binding interactions and improve BBB permeability [50,51]. Further modifications targeted key pharmacophoric regions of Montelukast. Amide modifications, including methylation and urea substitutions, were applied to enhance receptor selectivity and hydrogen bonding [52,53]. Carboxyl group modifications, including conversion to amides, esters, sulfonic acids, and hydroxamic acids, were performed to influence solubility, permeability, and metabolic stability [54,55]. Additional modifications, such as etherification, sulfonylation, and tertiary amine introduction, were employed to further optimize solubility and binding interactions [56,57]. Hybrid scaffold modifications were also explored, where fused ring systems and coumarin or chromene conjugations were introduced to enhance rigidity, metabolic stability, and receptor affinity [58,59]. Cyclization strategies, such as incorporating benzimidazole or quinazoline cores, were implemented to increase selectivity and interaction specificity [60,61] with DRD2 and 5-HT1A receptors. Each modified derivative was analyzed post-energy minimization to ensure structural integrity and feasibility for further molecular docking and simulation studies. The optimized structures were subsequently prepared for in silico screening to assess their potential as dual DRD2 antagonists and 5-HT1A agonists.

### 4.2. Three-Dimensional Structure Alignment and Similarity Analysis

To evaluate the structural consistency and conformational variations among Montelukast and its modified derivatives, 3D structure alignment was performed using the Superimpose feature in BIOVIA Discovery Studio version 2024 (Dassault Systèmes, Vélizy-Villacoublay, France) [62]. Due to the broad scope of the modification strategies applied in this study (including aromatic substitutions, heterocyclic replacements, amide modifications, halogenation, and other functional group alterations), the alignment process was conducted with a more flexible RMSD threshold. Unlike rigid structural modifications where strict RMSD values are enforced, this study acknowledged that significant variations in molecular geometry were expected. Therefore, the RMSD values were interpreted qualitatively rather than strictly, allowing for a broader range of conformational diversity while maintaining the core framework of Montelukast. Following structural alignment, a 3D similarity analysis was performed using the Activity Miner feature in Flare version 10.0 (Cresset BioMolecular Discovery Ltd., Litlington, UK) [63]. This analysis quantified the degree of similarity between Montelukast and its derivatives based on molecular surface properties, including electrostatic potential, hydrophobicity, and steric volume. Given the extensive modifications applied, the similarity coefficients were not constrained to stringent thresholds but rather used to categorize the modified structures into clusters with varying degrees of resemblance to the native Montelukast molecule. To further illustrate the similarity relationships among the modified derivatives, a similarity matrix was generated using the Tanimoto coefficient, which measures pairwise structural similarities based on molecular fingerprints. However, due to the diverse range of modifications, the Tanimoto coefficient values exhibited a wide distribution, reflecting the varying degrees of molecular transformation. The similarity data were processed and visualized as a heatmap graph using OriginLab Pro version 2024 (OriginLab Corporation, Northampton, MA, USA) [64]. The heatmap facilitated the identification of structurally related modifications while highlighting derivatives that introduced significant deviations. Additionally, to further explore the relationship between molecular similarity and docking outcomes, 3D box plot analyses were generated using Molecular Operating Environment (MOE) version 2024.06 (Chemical Computing Group, Montreal, QC, Canada) [65,66]. The plots were designed with the X-axis representing predicted activity ($PRED), the Y-axis indicating docking binding affinity ($FREE_BINDING_ENERGY), and the Z-axis displaying the statistical score of binding affinity ($Z-SCORE). With normalized axes activated, the primary focus of these 3D box plots was on free binding energy, enabling a detailed correlation between molecular modifications and docking performance. This analysis provided valuable insights into whether specific structural modifications enhanced or reduced binding affinity to the target receptors, helping to identify patterns in ligand–receptor interactions. By adopting a less stringent approach to RMSD and similarity coefficient thresholds, this comprehensive 3D alignment and similarity analysis facilitated the exploration of a broad range of molecular modifications while ensuring the retention of essential pharmacophoric features necessary for receptor interactions. The integration of heatmaps and 3D box plots further enhanced our ability to identify structure–activity relationships, ultimately guiding the selection of promising Montelukast derivatives for further investigation and potential therapeutic applications.

### 4.3. Molecular Docking Simulations and Binding Affinity Analysis

To gain deeper insights into the interactions between Montelukast and its modified derivatives with the DRD2 and 5-HT1A receptors, molecular docking simulations were performed. This study aimed to identify key binding residues involved in ligand–receptor interactions, analyze the types of intermolecular forces contributing to binding affinity, and explore the binding modes and orientations of the compounds. By evaluating these parameters, we sought to determine the structural modifications that enhance or diminish Montelukast’s ability to interact with these receptors. The 3D structures of DRD2 and 5-HT1A receptors were retrieved from the RCSB Protein Data Bank (PDB). The selection of these PDB models was based on structural resolution, functional relevance, and ligand-bound availability to ensure biologically meaningful docking studies. DRD2 was obtained with the PDB ID 6LUQ (chain A) at a resolution of 3.10 Å, while 5-HT1A was downloaded with the PDB ID 8FYX (chain D) at a resolution of 2.62 Å. Prior to docking simulations, receptor structures were refined using Swiss-PdbViewer version 4.1 (Swiss Institute of Bioinformatics, Lausanne, Switzerland) [67]. This refinement included energy minimization, removal of crystallographic water molecules, and correction of missing side chains, ensuring the receptor structures were in an optimal state for ligand docking (affina). The binding sites of the receptors were identified using PDBSum (European Bioinformatics Institute, Cambridge, UK) [68], a computational tool that provides detailed summaries of protein structures, including their secondary structural features and ligand-binding pockets. To validate the docking protocol, a redocking analysis was performed by redocking the co-crystallized ligands into their respective binding sites, and RMSD values were calculated. An RMSD below 2.0 Å confirmed the reliability of the docking approach. The binding site selection was guided by co-crystallized ligands and literature-reported key interaction residues for DRD2 and 5-HT1A, ensuring biologically relevant docking poses. This step was crucial in defining the receptor’s active site regions, ensuring that docking simulations were focused on biologically relevant interaction sites. Montelukast and its modified derivatives were generated and energy-minimized using the MM2 force field in Chem3D Ultra version 22 (PerkinElmer, Waltham, MA, USA). This minimization optimized the molecular geometries and ensured that each structure was in its lowest energy conformation before docking. Additionally, two standard ligands were included as reference compounds: Haloperidol, a well-known DRD2 antagonist, and Buspirone, a 5-HT1A agonist. These reference ligands underwent the same MM2 energy minimization to maintain consistency across all docking experiments.

Molecular docking simulations were conducted using the High Ambiguity Driven Protein–Protein Docking (HADDOCK) platform version 2.4 (University of Utrecht, Netherlands). HADDOCK is a widely used docking tool that employs ambiguous interaction restraints (AIRs) based on experimental or computationally derived interaction sites, allowing for improved docking accuracy [69,70]. Unlike traditional docking algorithms that rely solely on shape complementarity, HADDOCK integrates geometric constraints, energetic contributions, and experimental data, making it a powerful tool for ligand–protein interaction modeling [71]. The use of HADDOCK’s standalone interface allowed for advanced customization of docking parameters, incorporating both geometric and energetic restraints to enhance result reliability. To ensure that the best ligand–receptor complexes were selected for further analysis, two primary criteria were applied: (1) cluster population–docking models were grouped into clusters, with the highest populated cluster considered the most reliable; and (2) a larger cluster population indicates a greater degree of reproducibility and stability in the predicted binding mode. The HADDOCK score, which incorporates van der Waals energy, electrostatic interactions, desolvation energy, and buried surface area, was used to rank docking results. The model with the highest HADDOCK score was selected as the most favorable interaction, as it represents the strongest binding affinity. To refine binding energy predictions, PRODIGY (PROtein binDIng enerGY prediction) was employed. PRODIGY is a computational tool that estimates the binding free energy (Δ*G*) in kcal/mol based on the structural and energetic properties of ligand–receptor complexes. This calculation considers intermolecular contacts, desolvation effects, and buried surface area, providing a quantitative measure of binding affinity [72].

### 4.4. Molecular Dynamics (MD) Simulation for Structural Stability and Interaction Analysis

To gain deeper insights into the dynamic behavior, conformational stability, and molecular interactions of Montelukast and its derivatives in complex with target proteins, MD simulations were performed using GROMACS 2024.3 [73]. This simulation approach ensured a comprehensive analysis of ligand–receptor interactions over an extended timescale, allowing for precise evaluations of structural fluctuations and binding stability. For an accurate representation of ligand parameters, the General Amber Force Field (GAFF2) was applied, with atomic partial charges assigned using the AM1-BCC (Austin Model 1 with Bond Charge Corrections) method [74,75]. The *acpype* tool, integrated with AmberTools21, was used to prepare ligand topology files and parameterization. The receptor was modeled using the AMBER99sb force field, ensuring consistency in the treatment of bonded and non-bonded interactions. To simulate a realistic biological environment, the system was solvated using the SPC (Simple Point Charge) water model within a dodecahedral simulation box, maintaining a minimum buffer distance of 1.4 nm between the protein’s outermost atoms and the box boundaries. Periodic boundary conditions (PBCs) were applied in all directions to emulate bulk solvent effects. The system was neutralized by counterions, and physiological salt conditions were maintained by adding 100 mM NaCl to reflect in vivo ionic strength. Non-bonded interactions were handled with a 1.2 nm cutoff for van der Waals forces, while long-range electrostatics were computed using the Particle Mesh Ewald (PME) method to ensure computational efficiency and accuracy [76]. The system underwent an initial energy minimization using the steepest descent algorithm, reducing the maximum force threshold below 1000 kJ/mol/nm to eliminate steric clashes and unfavorable interactions. Equilibration was performed in two stages: NVT Ensemble (100 ps): The system was restrained while the Berendsen thermostat maintained a constant temperature of 310 K, allowing for gradual thermal stabilization. NPT Ensemble (100 ps): Pressure equilibration was carried out using the Berendsen barostat at 1 bar, ensuring appropriate system density adjustments. Following equilibration, a 100 ns production run was conducted under unrestrained conditions, with temperature and pressure regulation handled by the V-rescale thermostat and Parrinello–Rahman barostat, respectively. Throughout the simulation, coordinates were saved every 2 fs for detailed trajectory analysis. Key residues and intermolecular interactions were examined using molecular visualization tools, including PyMOL 3.1.3 (Schrödinger LLC, New York, NY, USA) [77], BIOVIA Discovery Studio 2024 (Dassault Systèmes, San Diego, CA, USA) [62], and UCSF Chimera 1.18 (UCSF, San Francisco, CA, USA) [78], allowing for detailed manual analysis and structural interpretation.

### 4.5. Molecular Mechanics/Poisson–Boltzmann Surface Area (MM/PBSA) Calculations

To quantify the binding free energy of Montelukast and its derivatives with the target receptor, the Molecular Mechanics/Poisson–Boltzmann Surface Area (MM/PBSA) method was employed in conjunction with MD simulations. This approach allows for an in-depth evaluation of ligand–receptor interactions by capturing both enthalpic and solvation contributions to binding affinity [79]. Representative snapshots from the MD trajectories were extracted to ensure a comprehensive sampling of protein conformations. Each selected frame underwent rigorous energy calculations, including gas-phase energy determination, solvation energy assessment using a continuum solvent model, and entropy estimation to account for molecular flexibility. In MM/PBSA modeling, the nonpolar solvation energy is assumed to be proportional to the solvent-accessible surface area, providing a refined representation of hydrophobic contributions to ligand binding. The Single-Trajectory Protocol (STP) was utilized to compute free energies, based on the assumption that minimal conformational changes occur upon ligand binding [80,81,82]. This approach simplifies energy estimations by deriving the ligand, receptor, and complex free energies from the same simulation trajectory. MM/PBSA calculations were performed using the gmx_MMPBSA module [83] integrated into the GROMACS simulation suite. The binding free energy (Δ*G_binding*) was determined using the following equation:

Δ*G_binding* = Δ*G_complex* − Δ*G_ligand* − Δ*G_receptor*

where

Δ*G_binding* represents the free energy associated with complex formation.
Δ*G_complex* is the total free energy of the solvated ligand–receptor complex.
Δ*G_ligand* corresponds to the free energy of the unbound ligand in solution.
Δ*G_receptor* denotes the free energy of the solvated unbound receptor.

### 4.6. Pharmacophore Modeling and In Silico ADMET Evaluation

To elucidate the key molecular features responsible for the binding interactions of Montelukast and its derivatives with target receptors, pharmacophore modeling was performed using LigandScout 4.5 (Inte:Ligand, Vienna, Austria) [84]. This software enables the construction of 3D pharmacophore models by identifying and mapping crucial chemical features, including hydrogen bond donors (HBDs), hydrogen bond acceptors (HBAs), hydrophobic regions, and electrostatic interaction sites. These features are essential for ligand recognition and contribute significantly to the binding affinity with target proteins. By employing LigandScout’s advanced feature recognition and alignment algorithms, this modeling approach facilitated the selection of promising Montelukast derivatives that exhibit favorable interaction profiles. Following the pharmacophore modeling, an in silico ADMET assessment was performed to evaluate the pharmacokinetic and toxicity profiles of Montelukast derivatives. SwissADME (Swiss Institute of Bioinformatics, Lausanne, Switzerland) [85] was utilized to predict physicochemical properties and drug-likeness based on established criteria such as molecular weight, lipophilicity (MlogP), HBD, HBA, and topological polar surface area (TPSA). These parameters were analyzed in accordance with Lipinski’s Rule of Five, a widely accepted guideline for assessing oral bioavailability. Compounds that adhered to these criteria were more likely to exhibit favorable absorption and permeability characteristics, making them suitable candidates for further investigation. Additionally, SwissADME provided insights into cytochrome P450 (CYP) enzyme inhibition, which is essential for understanding potential metabolic interactions and drug clearance rates. To further refine the selection of viable drug candidates, DataWarrior version 6.4.1 (OpenMolecules, Karlsruhe, Germany) [86] was employed to predict toxicity and drug-likeness. This computational tool integrates molecular descriptors and predictive algorithms to assess key toxicological endpoints, including mutagenicity, tumorigenicity, reproductive toxicity, and irritant properties. Drug-likeness scores were also calculated, providing an overall assessment of a compound’s suitability as a drug-like molecule. Compounds with high toxicity risks or poor drug-likeness scores were deprioritized to ensure the selection of safer and more effective derivatives.

## 5. Conclusions

In conclusion, this study highlights the rational modification of Montelukast to optimize its receptor interactions and explore its feasibility for drug repurposing in neuropsychiatric disorders. Computational molecular simulations identified MLK_MOD-42 and MLK_MOD-43 as the most promising derivatives. These compounds demonstrated higher predicted binding affinities toward DRD2 and 5-HT1A receptors, with MM/PBSA binding free energy values exceeding the native Montelukast. MLK_MOD-42 and MLK_MOD-43 were also predicted to have improved pharmacokinetic properties, including enhanced oral bioavailability, optimized lipophilicity, and reduced cytochrome P450 interactions, suggesting a lower risk of drug–drug interactions. However, these findings are based solely on in silico predictions and do not confirm improved efficacy or safety. While receptor binding affinity can indicate potential pharmacological activity, it does not always translate to in vivo therapeutic effects or reduced adverse outcomes. Toxicity predictions indicated a potentially lower likelihood of neuropsychiatric risks compared to the native compound, but these predictions require further experimental validation to confirm their clinical relevance. The structural modifications also contributed to a favorable balance between hydrophilic and hydrophobic interactions, which may enhance ligand stability within receptor binding sites. Importantly, this study did not evaluate potential off-target interactions, which could significantly impact the overall safety profile of these derivatives. Given their dual DRD2 antagonistic and 5-HT1A agonistic properties, these derivatives could serve as potential candidates for reducing neuropsychiatric side effects and warrant further investigations into drug repurposing for neuropsychiatric disorders. Nevertheless, this study does not establish definitive evidence of their safety or therapeutic benefits, as comprehensive in vitro and in vivo studies are required to assess their pharmacological profile, off-target interactions, and potential adverse effects before any clinical application can be considered.

## Figures and Tables

**Table 1:** Structural modifications of Montelukast: strategies, chemical changes, and predicted functional improvements.

Molecule	Modification Strategy	Modification	Purpose	2D Structure
MONTELUKAST (MLK)	Baseline	N/A	N/A	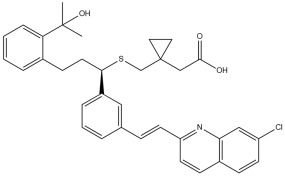
MLK_MOD_1	Aromatic Modification	Phenyl → Naphthyl	Increased π-π Stacking Interactions	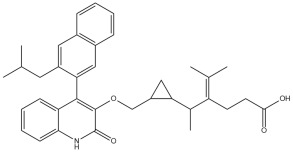
MLK_MOD_6	Heterocyclic Modification	Phenyl → Furan	Increased Polar Interactions	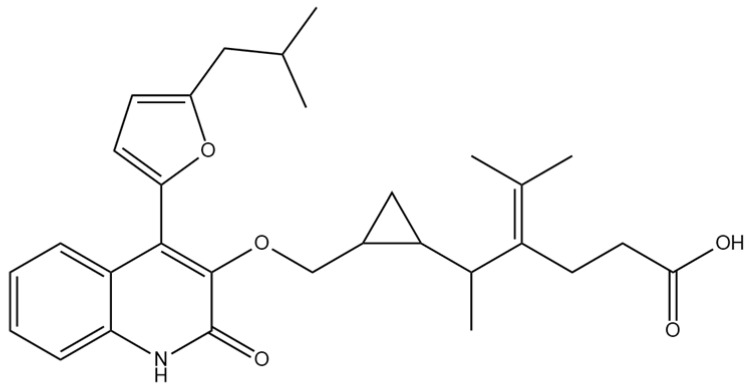
MLK_MOD_10	Carboxyl Modification	Carboxyl → Amide	Improved Permeability	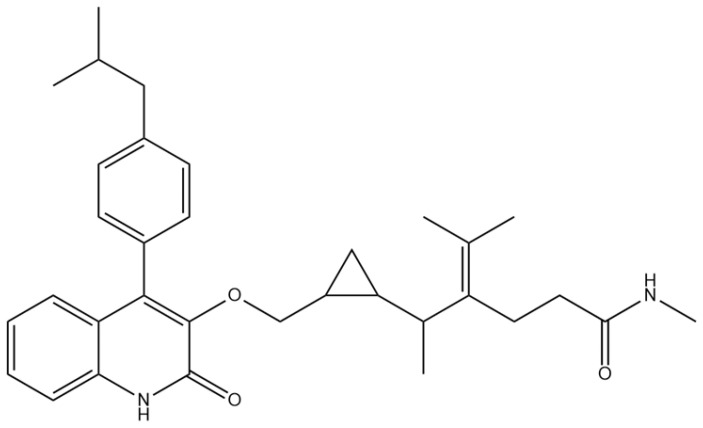
MLK_MOD_11	Carboxyl Modification	Carboxyl → Ester	Enhanced Lipophilicity	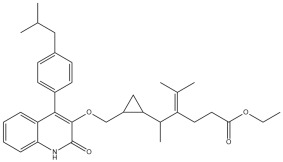
MLK_MOD_20	Basic Side Chain	Guanidine Addition	Increased Hydrogen Bonding	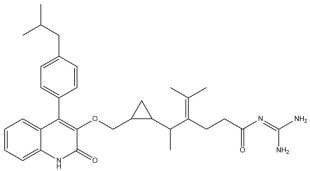
MLK_MOD_22	Heterocyclic Replacement	Phenyl → Quinoline	Improved Lipophilicity	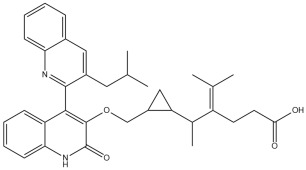
MLK_MOD_35	Basic Side Chain	Morpholine Addition	Improved Receptor Interactions	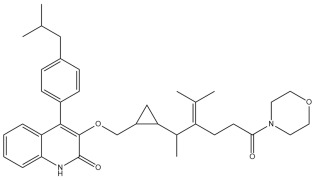
MLK_MOD_36	Basic Side Chain	Imidazole Addition	Increased H-bonding	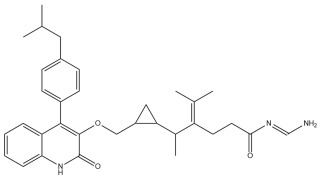
MLK_MOD_42	Fused Ring System	Phenyl → Fluorene	Increased Rigidity	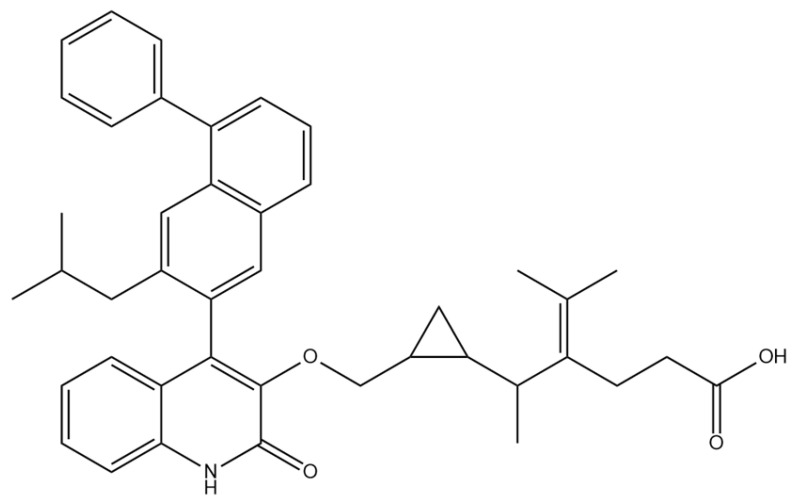
MLK_MOD_43	Fused Ring System	Phenyl → Carbazole	Increased Metabolic Stability	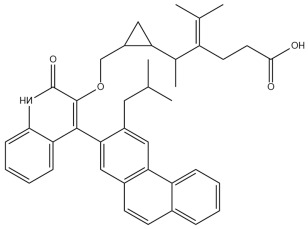
MLK_MOD_50	Hybrid Scaffold	Chromene Extension	Increased Hydrophobicity	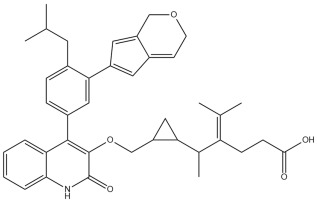

**Table 2:** Binding affinity and interaction energies of Haloperidol, Montelukast, and its best-performing modifications with the DRD2 receptor.

Complex	HADDOCK Score (a.u.)	Binding Energy (kcal/mol)	Van der Waals Energy	Electrostatic Energy	Desolvation Energy	RMSD
Haloperidol_DRD2 (Standard antagonist)	−41.0 ± 1.1	−9.82	−28.7 ± 0.8	−58.8 ± 3.9	−12.8 ± 0.4	0.2 ± 0.1
MLK_DDR2	−43.7 ± 4.7	−8.26	−36.1 ± 1.9	−25.1 ± 1.0	−11.7 ± 0.4	0.7 ± 0.0
MLK_MOD-43_DRD2	−46.0 ± 3.2	−11.22	−36.1 ± 2.2	−125.0 ± 13.2	−6.6 ± 0.9	0.3 ± 0.2
MLK_MOD-27_DRD2	−41.9 ± 0.7	−10.61	−33.7 ± 1.4	27.4 ± 13.2	−16.0 ± 1.4	0.6 ± 0.0
MLK_MOD-42_DRD2	−45.3 ± 0.9	−10.29	−40.5 ± 2.2	−25.8 ± 4.9	−9.4 ± 0.7	0.8 ± 0.0
MLK_MOD-22_DRD2	−38.4 ± 1.7	−10.11	−28.4 ± 1.9	−39.3 ± 10.9	−10.9 ± 0.8	1.0 ± 0.0
MLK_MOD-13_DRD2	−38.0 ± 2.2	−10.02	−29.6 ± 1.4	−61.9 ± 5.2	−7.6 ± 0.2	0.6 ± 0.0
MLK_MOD-33_DRD2	−42.1 ± 1.1	−9.97	−33.8 ± 0.5	12.2 ± 9.7	−13.2 ± 0.4	2.0 ± 0.0
MLK_MOD-3_DRD2	−38.3 ± 1.8	−9.91	−30.9 ± 0.8	−57.8 ± 9.0	−8.2 ± 0.2	0.7 ± 0.0
MLK_MOD-15_DRD2	−37.1 ± 2.5	−9.85	−31.0 ± 0.4	−49.5 ± 10.9	−7.3 ± 1.1	0.7 ± 0.0
MLK_MOD-36_DRD2	−37.9 ± 2.6	−9.79	−31.5 ± 1.8	−32.2 ± 7.8	−9.8 ± 0.3	0.8 ± 0.0
MLK_MOD-4_DRD2	−43.1 ± 2.5	−9.76	−35.1 ± 1.5	37.3 ± 6.3	−14.6 ± 0.7	0.4 ± 0.1

**Table 3:** Docking and binding energies of Montelukast (MLK) and its modifications against the 5-HT1A receptor.

Complex	HADDOCK Score (a.u.)	Binding Energy (kcal/mol)	Van der Waals Energy	Electrostatic Energy	Desolvation Energy	RMSD
Buspirone_5-HT1A (Standard agonist)	−23.1 ± 0.8	−8.3	−34.3 ± 0.3	−3.8 ± 2.4	−13.2 ± 0.1	0.2 ± 0.2
MLK_5-HT1A	−27.0 ± 1.3	−9.71	−28.7 ± 2.2	−49.5 ± 10.1	−11.8 ± 0.5	0.2 ± 0.2
MLK_MOD-24_5-HT1A	−34.2 ± 2.8	−11.04	−31.2 ± 0.6	−117.5 ± 6.1	−10.6 ± 0.8	0.3 ± 0.2
MLK_MOD-42_5-HT1A	−34.9 ± 1.6	−10.97	−38.2 ± 0.8	−2.2 ± 20.7	−17.4 ± 1.1	0.2 ± 0.1
MLK_MOD-21_5-HT1A	−34.1 ± 1.7	−10.76	−33.8 ± 0.6	−55.8 ± 0.3	−12.5 ± 0.9	0.3 ± 0.2
MLK_MOD-1_5-HT1A	−31.6 ± 0.6	−10.74	−31.1 ± 2.0	−46.7 ± 6.4	−14.3 ± 0.4	0.4 ± 0.2
MLK_MOD-12_5-HT1A	−34.9 ± 2.3	−10.64	−30.9 ± 0.8	−84.8 ± 7.3	−9.4 ± 0.3	0.2 ± 0.2
MLK_MOD-2_5-HT1A	−32.0 ± 1.7	−10.45	−32.5 ± 1.5	−16.9 ± 3.5	−12.4 ± 0.3	0.4 ± 0.2
MLK_MOD-16_5-HT1A	−31.8 ± 1.0	−10.44	−31.5 ± 0.8	−22.6 ± 0.6	−12.7 ± 0.2	0.2 ± 0.1
MLK_MOD-38_5-HT1A	−32.3 ± 0.7	−10.43	−33.3 ± 1.1	−37.9 ± 5.4	−14.2 ± 1.1	0.4 ± 0.2
MLK_MOD-43_5-HT1A	−31.3 ± 1.6	−10.42	−30.2 ± 1.4	−21.1 ± 0.5	−11.2 ± 0.1	0.1 ± 0.0
MLK_MOD-41_5-HT1A	−30.4 ± 1.0	−10.40	−31.3 ± 0.7	−13.5 ± 3.6	−12.1 ± 0.9	0.6 ± 0.0

**Table 4:** Molecular interaction profiles of Montelukast (MLK) modifications with DRD2 and 5-HT1A receptors compared to standard ligands.

Complex	CC	CO	CN	CX	OO	OX	NO	NN	NX	XX
Haloperidol_DRD2 (Standard antagonist)	2782	945	700	173	61	42	79	25	38	1
MLK_DDR2	1985	751	624	70	59	20	72	13	22	0
MLK_MOD-43_DRD2	3193	1393	1118	22	100	2	123	25	0	0
MLK_MOD-27_DRD2	3813	1400	1074	315	105	77	159	48	74	4
MLK_MOD-42_DRD2	3427	1349	1071	3	101	2	103	24	2	0
MLK_MOD-22_DRD2	3183	1299	881	17	115	5	135	37	0	0
MLK_MOD-13_DRD2	2796	1124	865	138	93	32	102	28	27	1
Buspirone_5-HT1A (Standard agonist)	2650	888	1268	42	63	2	217	141	10	0
MLK_5-HT1A	2636	1001	730	127	81	34	77	12	19	2
MLK_MOD-24_5-HT1A	3298	1293	1017	57	109	6	154	53	3	0
MLK_MOD-42_5-HT1A	4104	1407	1090	63	84	3	96	22	2	0
MLK_MOD-21_5-HT1A	3558	1471	1028	66	117	5	159	48	3	0
MLK_MOD-1_5-HT1A	3568	1345	953	71	113	2	115	22	2	0
MLK_MOD-43_5-HT1A	3524	1252	946	50	76	5	98	28	1	0

**Table 5:** Molecular dynamics analysis of ligand–glucocorticoid receptor (GR) complexes: structural stability and hydrogen bond interactions.

Complex	Average RMSD (Å)	Average RMSF (Å)	Average RoG (Å)	Number of Hydrogen Bonds Between the Ligand and Receptor
DRD2 (Apo-protein)	1.806	0.662	2.105	N/A
Haloperidol_DRD2 (Standard antagonist)	2.776	1.708	2.643	2
MLK_DDR2	2.635	1.375	2.554	0
MLK_MOD-43_DRD2	2.441	1.954	2.831	3
MLK_MOD-27_DRD2	2.674	1.743	2.789	2
MLK_MOD-42_DRD2	2.145	1.811	2.815	5
MLK_MOD-22_DRD2	2.262	1.429	2.524	2
MLK_MOD-13_DRD2	2.517	1.465	2.483	4
5-HT1A (Apo-protein)	2.011	1.012	2.345	N/A
Buspirone_5-HT1A (Standard agonist)	2.487	1.440	2.712	1
MLK_5-HT1A	2.234	1.488	2.862	4
MLK_MOD-24_5-HT1A	2.331	1.299	2.847	3
MLK_MOD-42_5-HT1A	2.189	1.129	2.803	2
MLK_MOD-21_5-HT1A	2.446	1.315	2.818	1
MLK_MOD-1_5-HT1A	2.284	1.421	2.805	3
MLK_MOD-43_5-HT1A	2.221	1.319	2.916	2

**Table 6:** MM/PBSA free binding energy of Montelukast (MLK) modifications with DRD2 and 5-HT1A receptors.

Complex	MM/PBSA Free Binding Energy Δ*G_binding* (kcal/mol)
Haloperidol_DRD2 (standard antagonist)	−23.41 ± 3.24
MLK_DDR2	−19.32 ± 4.18
MLK_MOD-43_DRD2	−27.37 ± 2.22
MLK_MOD-27_DRD2	−25.65 ± 3.01
MLK_MOD-42_DRD2	−31.92 ± 2.54
MLK_MOD-22_DRD2	−26.81 ± 3.32
MLK_MOD-13_DRD2	−24.15 ± 2.38
Buspirone_5-HT1A (standard agonist)	−27.92 ± 1.34
MLK_5-HT1A	−20.14 ± 3.67
MLK_MOD-24_5-HT1A	−24.90 ± 2.33
MLK_MOD-42_5-HT1A	−30.22 ± 2.29
MLK_MOD-21_5-HT1A	−27.87 ± 3.38
MLK_MOD-1_5-HT1A	−22.82 ± 2.29
MLK_MOD-43_5-HT1A	−28.19 ± 2.14

**Table 7:** ADME properties and CYP inhibition profiles of Montelukast (MLK) and its derivatives.

Molecule	MW (g/mol)	MlogP	HBA	HBD	TPSA (Å2)	Lipinski Violation	CYP Inhibitor
Montelukast (MLK)	586.18	5.70	4	2	95.72	2 violations: MW > 500, MLOGP > 4.15	CYP2C19, CYP2D6, CYP3A4
MLK_MOD-1	551.72	5.65	4	2	79.39	2 violations: MW > 500, MLOGP > 4.15	CYP2C19, CYP2D6, CYP3A4
MLK_MOD-6	491.62	3.94	5	2	92.53	0	CYP2C19, CYP2C9, CYP2D6, CYP3A4
MLK_MOD-12	517.66	4.55	5	3	99.62	2 violations: MW > 500, MLOGP > 4.15	CYP2C19, CYP2C9, CYP2D6, CYP3A4
MLK_MOD-13	519.65	5.46	5	2	79.39	2 violations: MW > 500, MLOGP > 4.15	CYP2C19, CYP2C9, CYP2D6, CYP3A4
MLK_MOD-21	552.70	4.69	5	2	92.28	2 violations: MW > 500, MLOGP > 4.15	CYP2C19, CYP2C9, CYP2D6, CYP3A4
MLK_MOD-22	552.70	4.93	5	2	92.28	2 violations: MW > 500, MLOGP > 4.15	CYP2C19, CYP2C9, CYP2D6, CYP3A4
MLK_MOD-35	570.76	4.61	4	1	71.63	2 violations: MW > 500, MLOGP > 4.15	CYP2C19, CYP2D6, CYP3A4
MLK_MOD-36	527.70	5.21	4	2	97.54	2 violations: MW > 500, MLOGP > 4.15	CYP2C19, CYP2C9, CYP2D6, CYP3A4
MLK_MOD-42	627.81	6.49	4	2	79.39	2 violations: MW > 500, MLOGP > 4.15	CYP2D6
MLK_MOD-43	601.77	6.18	4	2	79.39	2 violations: MW > 500, MLOGP > 4.15	CYP2D6

**Table 8:** Predicted drug-likeness and toxicity profiles of Montelukast (MLK) derivatives.

Molecule	Drug-Likeness	Mutagenic	Tumorigenic	Reproductive Effective	Irritant
MLK_MOD-1	1.433	None	None	High	None
MLK_MOD-6	2.669	High	None	None	None
MLK_MOD-12	1.433	None	None	High	None
MLK_MOD-13	0.093	None	None	High	None
MLK_MOD-21	2.031	None	None	None	None
MLK_MOD-22	1.433	None	None	None	None
MLK_MOD-35	3.371	None	None	High	High
MLK_MOD-36	1.504	None	None	High	None
MLK_MOD-42	4.862	None	None	High	None
MLK_MOD-43	1.433	None	None	High	None

## Data Availability

The original contributions presented in this study are included in the article/Supplementary Material. Further inquiries can be directed to the corresponding author(s).

## Supplementary Materials

The following supporting information can be downloaded at https://www.mdpi.com/article/10.3390/ph18040559/s1. Supplementary Data S1: Montelukast modifications strategy; Supplementary Data S2: Complete docking and binding energy results of Montelukast and its modifications against the DRD2 and 5-HT1A receptors; Supplementary Data S3: Molecular interaction profiles of Montelukast modifications with DRD2 and 5-HT1A receptors; Supplementary Data S4: ADME properties and CYP inhibition profiles of Montelukast and its derivatives; Supplementary Data S5: Predicted drug-likeness and toxicity profiles of Montelukast derivatives.

## Abbreviations

The following abbreviations are used in this manuscript:

5-HT1A	Serotonin 1A receptor
ADMET	Absorption, distribution, metabolism, excretion, and toxicity
AIRs	Ambiguous interaction restraints
BBB	Blood–brain barrier
CASTp	Computed atlas of surface topography of proteins
CNS	Central nervous system
CYP	Cytochrome P450
DRD2	D2 dopamine receptor
FDA	Food and Drug Administration
GAFF2	General amber force field
HADDOCK	High ambiguity driven protein–protein docking
HBA	Hydrogen bond acceptor
HBD	Hydrogen bond donor
IND	Investigational new drug
LTRA	Leukotriene receptor antagonist
MD	Molecular dynamics
MLK	Montelukast
MM/PBSA	Molecular mechanics/Poisson–Boltzmann surface area
MOE	Molecular operating environment
NPAEs	Neuropsychiatric adverse events
NPT	Number of particles, pressure, and temperature
NVT	Number of particles, volume, and temperature
PME	Particle mesh Ewald
PRODIGY	Protein binding energy prediction
RMSD	Root mean square deviation
RMSF	Root mean square fluctuation
RoG	Radius of gyration
SPCE	Single point charge extended
SPR	Surface plasmon resonance
STP	Single-trajectory protocol
TPSA	Topological polar surface area
