# Supplementary material for: Computational Investigation of Montelukast and Its Structural Derivatives for Binding Affinity to Dopaminergic and Serotonergic Receptors: Insights from a Comprehensive Molecular Simulation
Source: Pharmaceuticals (Basel). 2025 Apr 10;18(4):559. doi: 10.3390/ph18040559 (PMC12030116; doi:10.3390/ph18040559)
Supplement: Supplementary file 1 [file pharmaceuticals-18-00559-s001.zip › Supplementary Data S1 - Montelukast Modifications Strategy.pdf]

SUPPLEMENTARY DATA S1

MONTELUKAST (MLK) MODIFICATIONS STRATEGY

Molecule	Modification Strategy	Modification	Purpose	2D Structure
MONTELUKAST (MLK)	Baseline	N/A	N/A	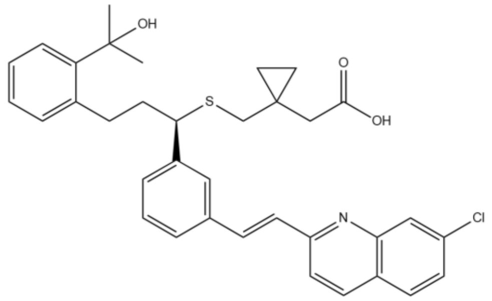
MLK_MOD_1	Aromatic Modification	Phenyl → Naphthyl	Increased π-π stacking interactions	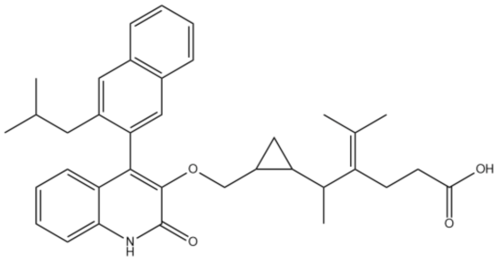
MLK_MOD_2	Aromatic Modification	Phenyl → Biphenyl	Increased hydrophobicity	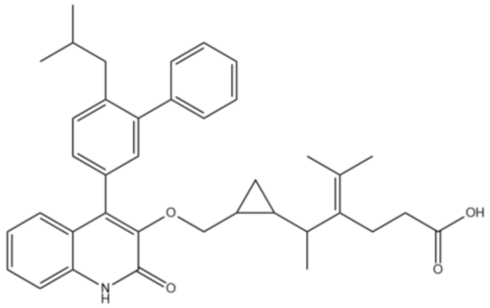
MLK_MOD_3	Aromatic Modification	Phenyl → Fluorophenyl	Improved receptor binding	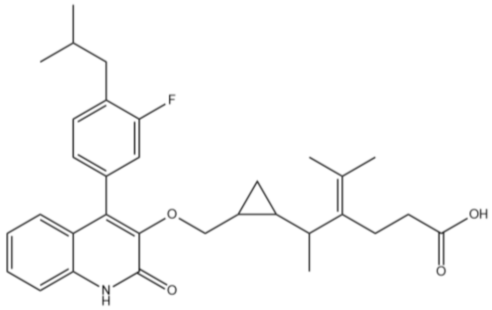
MLK_MOD_4	Heterocyclic Modification	Phenyl → Pyridine	Enhanced H-bonding	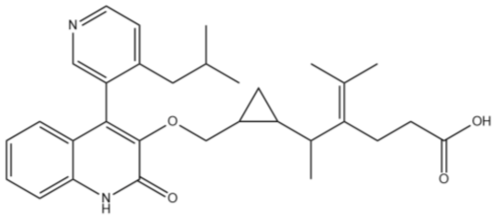

MLK_MOD_5	Heterocyclic Modification	Phenyl → Thiophene	Improved metabolic stability	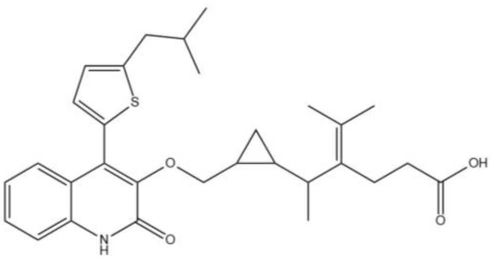
MLK_MOD_6	Heterocyclic Modification	Phenyl → Furan	Increased polar interactions	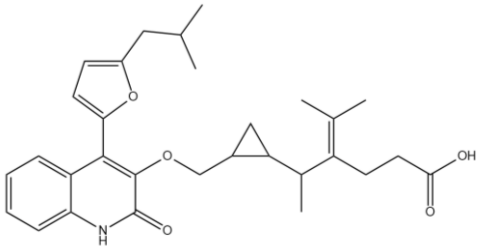
MLK_MOD_7	Aromatic Extension	Phenyl → Benzodioxole	Enhanced receptor affinity	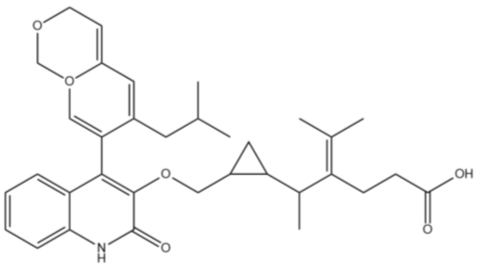
MLK_MOD_8	Amide Modification	Methylation at Amide Nitrogen	Increased receptor selectivity	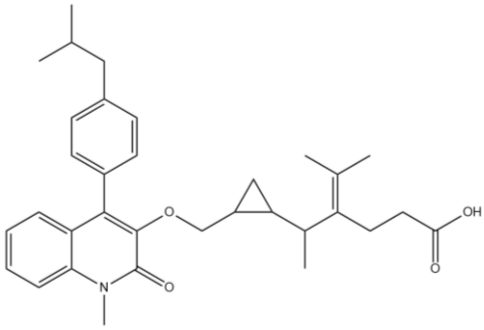
MLK_MOD_9	Amide Modification	Amide → Urea	Enhanced hydrogen bonding	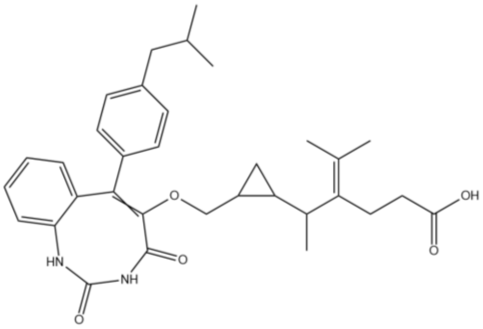
MLK_MOD_10	Carboxyl Modification	Carboxyl → Amide	Improved permeability	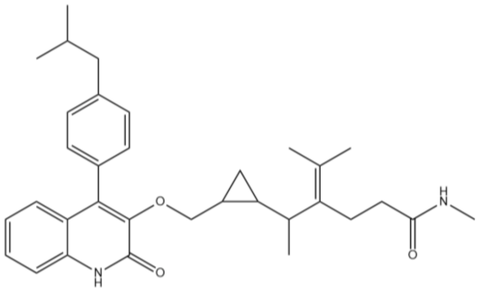

MLK_MOD_11	Carboxyl Modification	Carboxyl → Ester	Enhanced lipophilicity	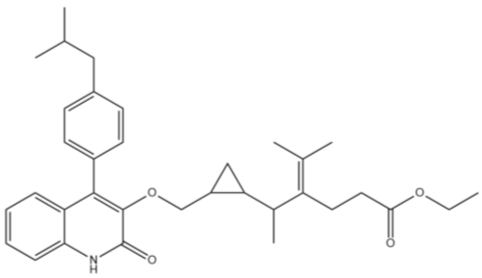
MLK_MOD_12	Hydroxylation	Hydroxyl Addition (at Phenyl)	Improved water solubility	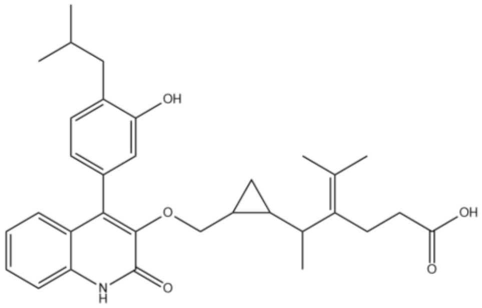
MLK_MOD_13	Halogenation	Fluorine Addition (at Aromatic Ring)	Increased metabolic stability	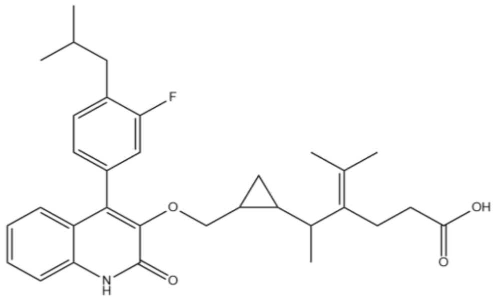
MLK_MOD_14	Halogenation	Chlorine Addition (at Aromatic Ring)	Enhanced lipophilicity	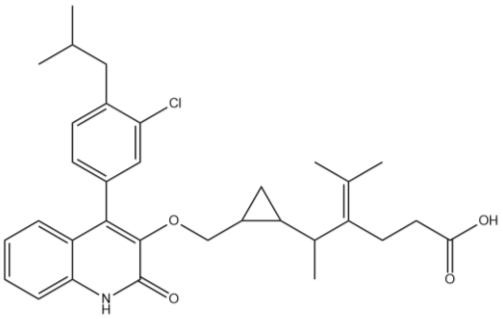
MLK_MOD_15	Etherification	Methoxy Addition (at Aromatic Ring)	Increased receptor binding	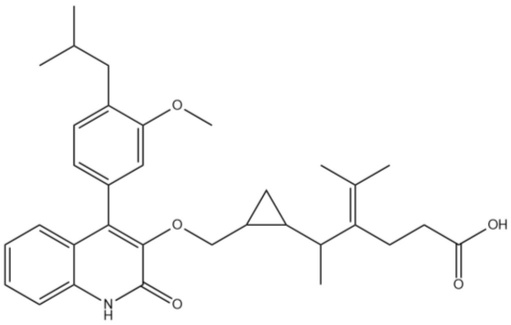

MLK_MOD_16	Fluorinated Modification	Trifluoromethyl Addition (at Aromatic Ring)	Improved BBB permeability	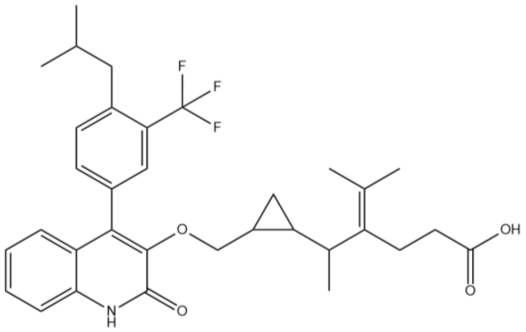
MLK_MOD_17	Sulfonylation	Sulfonamide Addition	Improved hydrophilicity	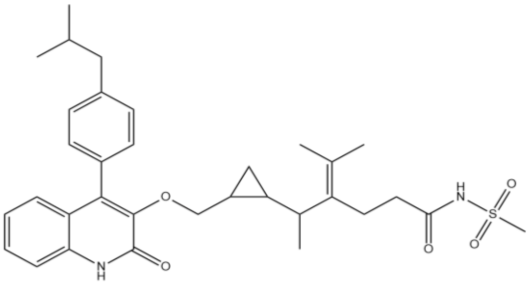
MLK_MOD_18	Etherification	Ether Addition (at Alkyl Chain)	Increased solubility	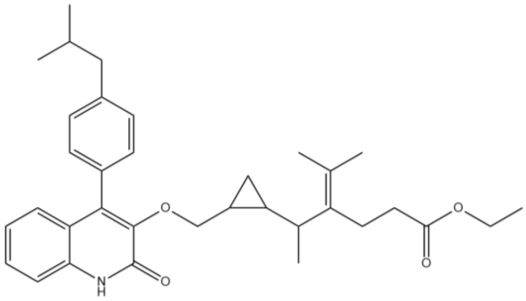
MLK_MOD_19	Amination	Tertiary Amine Addition	Enhanced receptor affinity	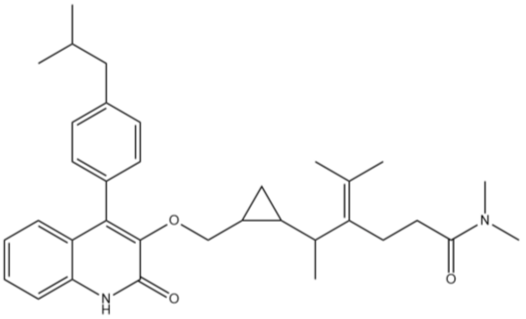
MLK_MOD_20	Basic Side Chain	Guanidine Addition	Increased hydrogen bonding	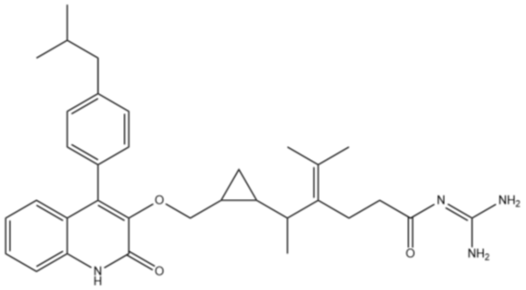
MLK_MOD_21	Heterocyclic Replacement	Phenyl → Indole	Increased π-π stacking interactions	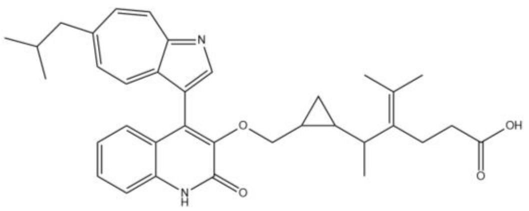

MLK_MOD_22	Heterocyclic Replacement	Phenyl → Quinoline	Improved lipophilicity	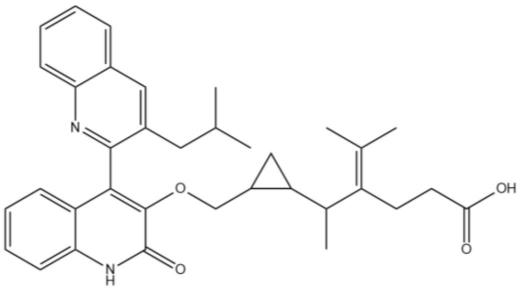
MLK_MOD_23	Heterocyclic Replacement	Phenyl → Isoquinoline	Enhanced receptor interactions	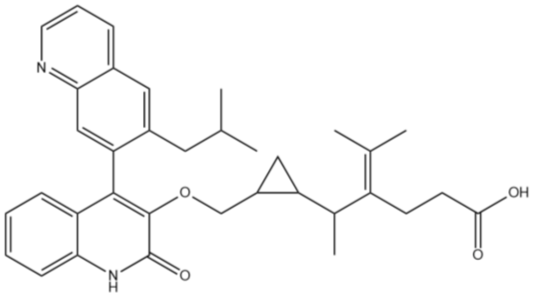
MLK_MOD_24	Heterocyclic Replacement	Phenyl → Benzimidazole	Stronger H-bonding	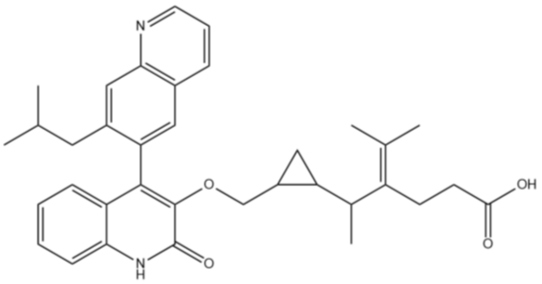
MLK_MOD_25	Heterocyclic Replacement	Phenyl → Benzothiazole	Increased metabolic stability	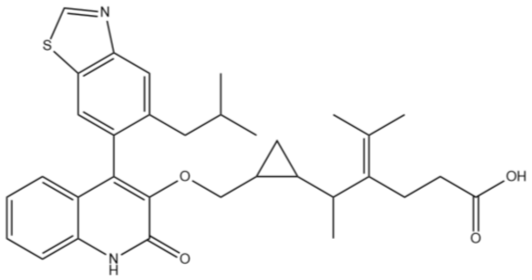
MLK_MOD_26	Heterocyclic Replacement	Phenyl → Benzoxazole	Increased polarity	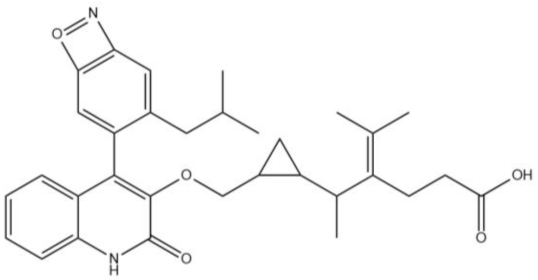

MLK_MOD_27	Fluorination	CF3 Addition (at Benzimidazole)	Enhanced BBB permeability	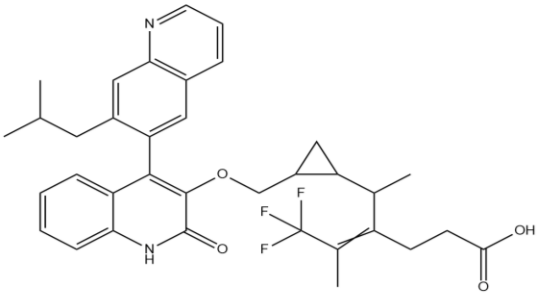
MLK_MOD_28	Halogenation	Br Addition (at Phenyl)	Increased hydrophobic interactions	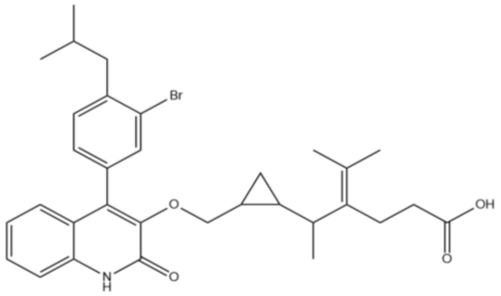
MLK_MOD_29	Carboxyl Replacement	Carboxyl → Sulfonic Acid	Increased solubility	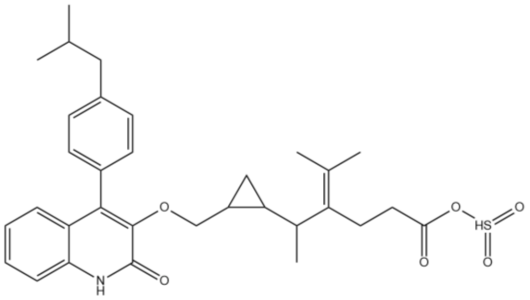
MLK_MOD_30	Carboxyl Replacement	Carboxyl → Hydroxamic Acid	Metal chelation properties	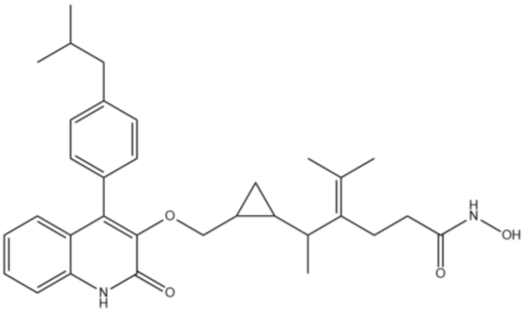
MLK_MOD_31	Etherification	Benzyl Ether Addition	Enhanced receptor binding	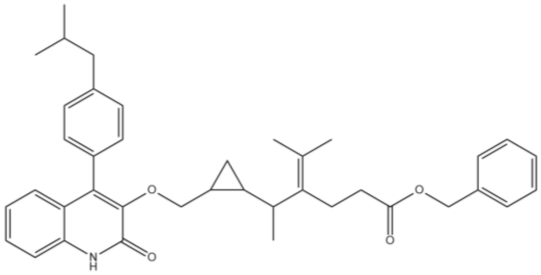

MLK_MOD_32	Amide Modification	N-Benzyl Amide	Improved selectivity	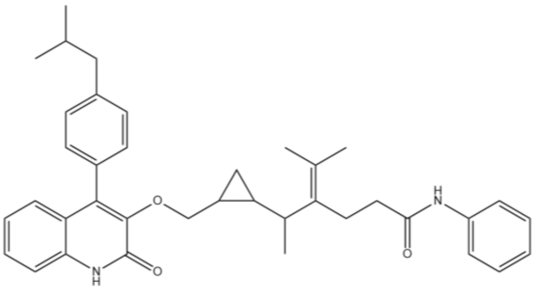
MLK_MOD_33	Heterocyclic Amide	Pyrrolidinone Amide	Increased polarity	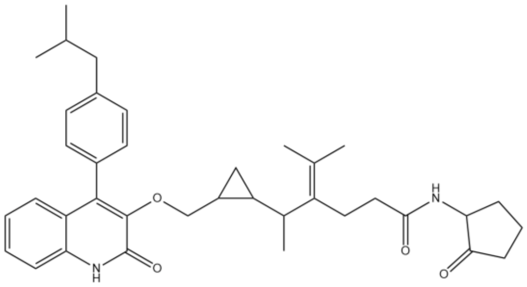
MLK_MOD_34	Basic Side Chain	Piperazine Addition	Enhanced binding affinity	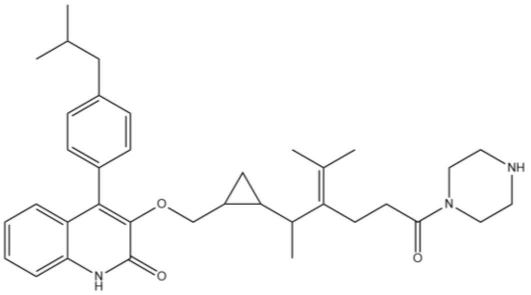
MLK_MOD_35	Basic Side Chain	Morpholine Addition	Improved receptor interactions	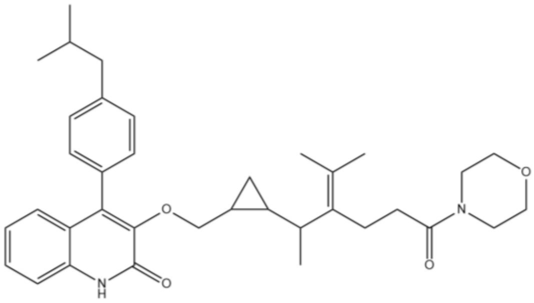
MLK_MOD_36	Basic Side Chain	Imidazole Addition	Increased H-bonding	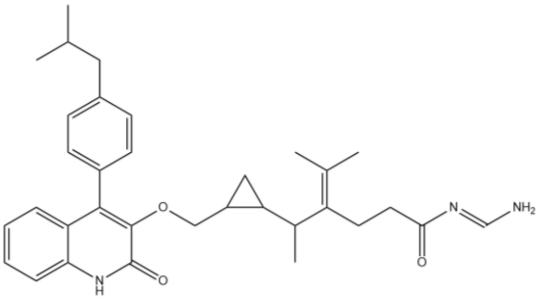

MLK_MOD_37	Hydrophobic Tail Modification	Longer Alkyl Chain	Improved lipophilicity	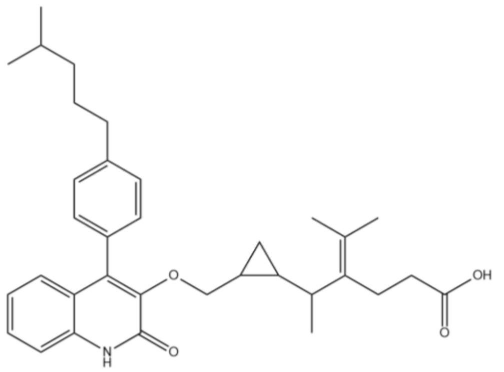
MLK_MOD_38	Hydrophobic Tail Modification	Cyclohexyl Tail	Increased rigidity	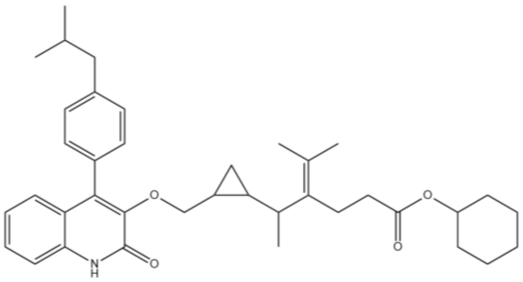
MLK_MOD_39	Sulfur Addition	Thiourea Group	Enhanced interactions	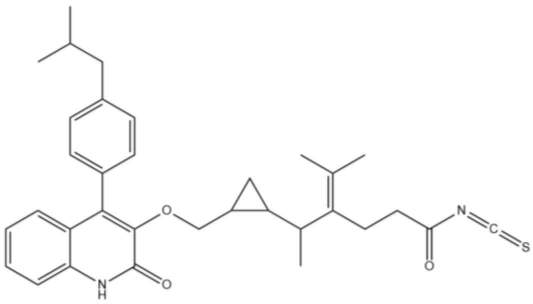
MLK_MOD_40	Hydrophilic Addition	PEGylation	Increased solubility	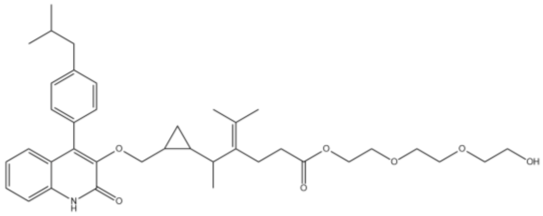
MLK_MOD_41	Aromatic Expansion	Biphenyl Extension	Enhanced π-π stacking	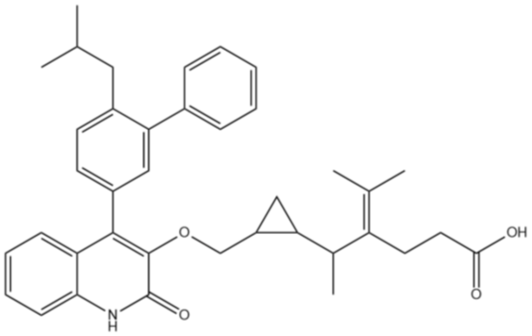

MLK_MOD_42	Fused Ring System	Phenyl → Fluorene	Increased rigidity	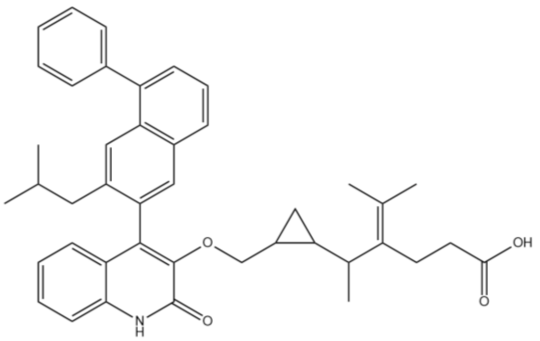
MLK_MOD_43	Fused Ring System	Phenyl → Carbazole	Increased metabolic stability	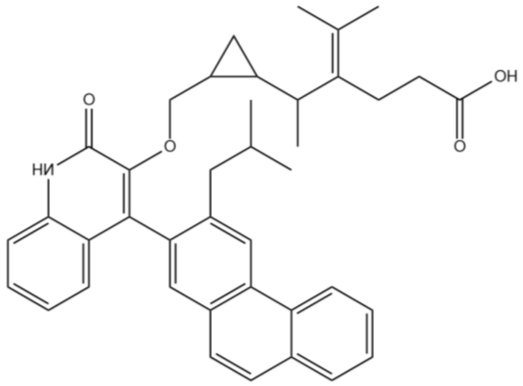
MLK_MOD_44	Heteroaromatic Substitution	Phenyl → Thiophene	Increased lipophilicity	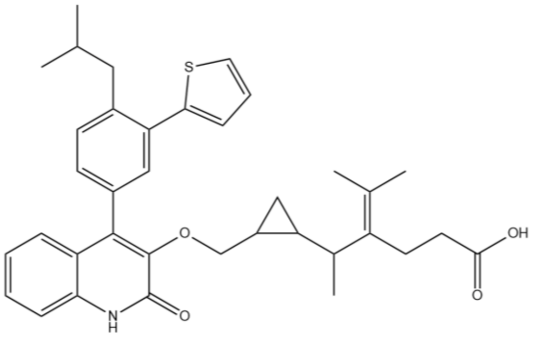
MLK_MOD_45	Heteroaromatic Substitution	Phenyl → Furan	Improved hydrogen bonding	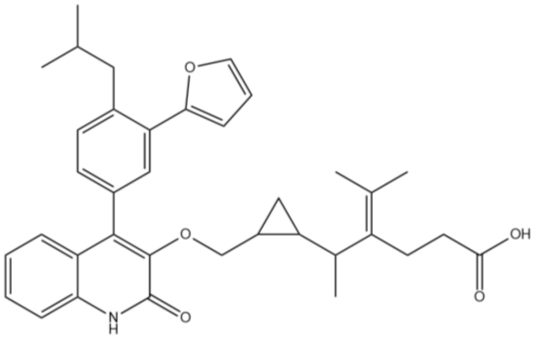
MLK_MOD_46	Heteroaromatic Substitution	Phenyl → Pyridine	Improved solubility	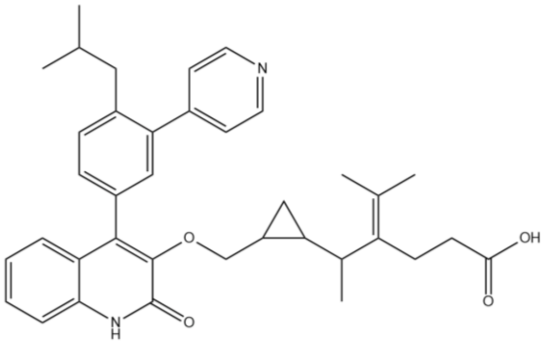

MLK_MOD_47	Ring Closure	Quinazoline Core	Increased selectivity	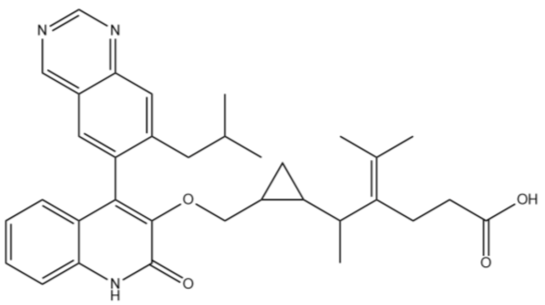
MLK_MOD_48	Ring Closure	Benzimidazole Core	Increased rigidity	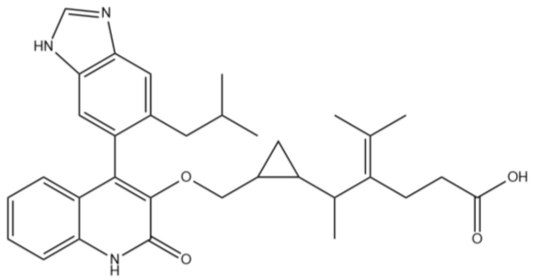
MLK_MOD_49	Hybrid Scaffold	Coumarin-Conjugation	Improved metabolic stability	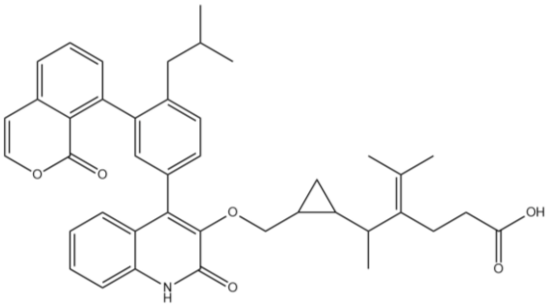
MLK_MOD_50	Hybrid Scaffold	Chromene Extension	Increased hydrophobicity	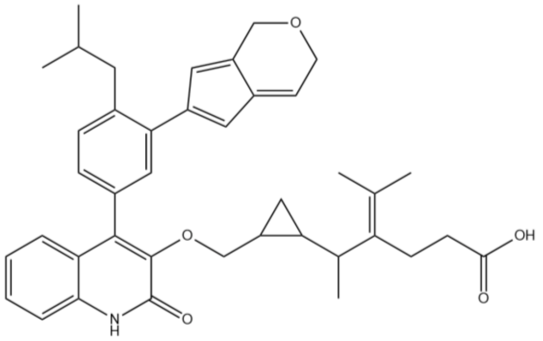
