# Supplementary material for: Computational Investigation of Montelukast and Its Structural Derivatives for Binding Affinity to Dopaminergic and Serotonergic Receptors: Insights from a Comprehensive Molecular Simulation
Source: Pharmaceuticals (Basel). 2025 Apr 10;18(4):559. doi: 10.3390/ph18040559 (PMC12030116; doi:10.3390/ph18040559)
Supplement: Supplementary file 1 [file pharmaceuticals-18-00559-s001.zip › Supplementary Data S4 - ADME properties and CYP inhibition profiles of Montelukast and its derivatives.pdf]

SUPPLEMENTARY DATA S4

IN SILICO ADME EVALUATION

Molecule	MW (g/mol)	MlogP	HBA	HBD	TPSA (Å2)	Lipinski Violation	CYP Inhibitor
Montelukast (MLK)	586.18	5.70	4	2	95.72	2 violations: MW >500, MLOGP >4.15	CYP2C19, CYP2D6, CYP3A4
MLK_MOD-1	551.72	5.65	4	2	79.39	2 violations: MW >500, MLOGP >4.15	CYP2C19, CYP2D6, CYP3A4
MLK_MOD-2	577.75	5.97	4	2	79.39	2 violations: MW >500, MLOGP >4.15	CYP2C19, CYP2D6, CYP3A4
MLK_MOD-3	519.65	5.46	5	2	79.39	2 violations: MW >500, MLOGP >4.15	CYP2C19, CYP2C9, CYP2D6, CYP3A4
MLK_MOD-4	502.64	4.10	5	2	92.28	1 violation: MW >500	CYP2C19, CYP2C9, CYP2D6, CYP3A4
MLK_MOD-5	507.68	4.75	4	2	107.63	2 violations: MW >500, MLOGP >4.15	CYP2C19, CYP2C9, CYP2D6, CYP3A4
MLK_MOD-6	491.62	3.94	5	2	92.53	0	CYP2C19, CYP2C9, CYP2D6, CYP3A4
MLK_MOD-7	560.70	4.26	6	2	97.85	2 violations: MW >500, MLOGP >4.15	CYP2C9, CYP3A4
MLK_MOD-8	515.68	5.28	4	1	68.53	2 violations: MW >500, MLOGP >4.15	CYP2C19, CYP2C9, CYP2D6, CYP3A4
MLK_MOD-9	544.68	4.75	5	3	112.25	2 violations: MW >500, MLOGP >4.15	CYP2C9, CYP2D6, CYP3A4
MLK_MOD-10	514.70	4.88	3	2	71.19	2 violations: MW >500, MLOGP >4.15	CYP2C19, CYP2D6, CYP3A4

MLK_MOD-11	529.71	5.47	4	1	68.39	2 violations: MW >500, MLOGP >4.15	CYP2C19, CYP2D6, CYP3A4
MLK_MOD-12	517.66	4.55	5	3	99.62	2 violations: MW >500, MLOGP >4.15	CYP2C19, CYP2C9, CYP2D6, CYP3A4
MLK_MOD-13	519.65	5.46	5	2	79.39	2 violations: MW >500, MLOGP >4.15	CYP2C19, CYP2C9, CYP2D6, CYP3A4
MLK_MOD-14	536.10	5.55	4	2	79.39	2 violations: MW >500, MLOGP >4.15	CYP2C19, CYP2C9, CYP2D6, CYP3A4
MLK_MOD-15	531.68	4.74	5	2	88.62	2 violations: MW >500, MLOGP >4.15	CYP2C19, CYP2C9, CYP2D6, CYP3A4
MLK_MOD-16	569.65	5.82	7	2	79.39	2 violations: MW >500, MLOGP >4.15	CYP2D6, CYP3A4
MLK_MOD-17	578.76	3.94	5	2	113.71	1 violation: MW >500	CYP2C19, CYP2C9, CYP2D6, CYP3A4
MLK_MOD-18	529.71	5.47	4	1	68.39	2 violations: MW >500, MLOGP >4.15	CYP2C19, CYP2D6, CYP3A4
MLK_MOD-19	528.72	5.06	3	1	62.40	2 violations: MW >500, MLOGP >4.15	CYP2C19, CYP2D6, CYP3A4
MLK_MOD-20	542.71	4.82	4	3	123.56	2 violations: MW >500, MLOGP >4.15	CYP2C9, CYP2D6, CYP3A4
MLK_MOD-21	552.70	4.69	5	2	92.28	2 violations: MW >500, MLOGP >4.15	CYP2C19, CYP2C9, CYP2D6, CYP3A4
MLK_MOD-22	552.70	4.93	5	2	92.28	2 violations: MW >500, MLOGP >4.15	CYP2C19, CYP2C9, CYP2D6, CYP3A4
MLK_MOD-23	552.70	4.93	5	2	92.28	2 violations: MW >500,	CYP2C19, CYP2C9,

						MLOGP >4.15	CYP2D6, CYP3A4
MLK_MOD-24	552.70	4.93	5	2	92.28	2 violations: MW >500, MLOGP >4.15	CYP2C19, CYP2C9, CYP2D6, CYP3A4
MLK_MOD-25	558.73	4.87	5	2	120.52	2 violations: MW >500, MLOGP >4.15	CYP2C9, CYP2D6, CYP3A4
MLK_MOD-26	529.65	4.76	6	2	108.82	2 violations: MW >500, MLOGP >4.15	CYP2C9
MLK_MOD-27	606.67	5.20	8	2	92.28	2 violations: MW >500, MLOGP >4.15	CYP2D6, CYP3A4
MLK_MOD-28	580.55	5.64	4	2	79.39	2 violations: MW >500, MLOGP >4.15	CYP2C19, CYP2D6, CYP3A4
MLK_MOD-29	565.72	4.97	6	1	141.33	2 violations: MW >500, MLOGP >4.15	CYP2C9
MLK_MOD-30	516.67	4.69	4	3	91.42	2 violations: MW >500, MLOGP >4.15	CYP2C19, CYP2C9, CYP2D6, CYP3A4
MLK_MOD-31	591.78	6.14	4	1	68.39	2 violations: MW >500, MLOGP >4.15	CYP2C19, CYP2D6, CYP3A4
MLK_MOD-32	576.77	5.83	3	2	71.19	2 violations: MW >500, MLOGP >4.15	CYP2C19, CYP2D6, CYP3A4
MLK_MOD-33	582.77	4.71	4	2	88.26	2 violations: MW >500, MLOGP >4.15	CYP2C19, CYP2D6, CYP3A4
MLK_MOD-34	569.78	4.61	4	2	74.43	2 violations: MW >500, MLOGP >4.15	CYP2C19, CYP2D6, CYP3A4
MLK_MOD-35	570.76	4.61	4	1	71.63	2 violations: MW >500, MLOGP >4.15	CYP2C19, CYP2D6, CYP3A4

MLK_MOD-36	527.70	5.21	4	2	97.54	2 violations: MW >500, MLOGP >4.15	CYP2C19, CYP2C9, CYP2D6, CYP3A4
MLK_MOD-37	529.71	5.47	4	2	79.39	2 violations: MW >500, MLOGP >4.15	CYP2C19, CYP2C9, CYP2D6, CYP3A4
MLK_MOD-38	583.80	6.18	4	1	68.39	2 violations: MW >500, MLOGP >4.15	CYP2D6, CYP3A4
MLK_MOD-39	542.73	6.72	4	1	103.61	2 violations: MW >500, MLOGP >4.15	CYP2C19, CYP2C9, CYP3A4
MLK_MOD-40	633.81	3.78	7	2	107.08	1 violation: MW >500	CYP2D6, CYP3A4
MLK_MOD-41	577.75	5.97	4	2	79.39	2 violations: MW >500, MLOGP >4.15	CYP2C19, CYP2D6, CYP3A4
MLK_MOD-42	627.81	6.49	4	2	79.39	2 violations: MW >500, MLOGP >4.15	CYP2D6
MLK_MOD-43	601.77	6.18	4	2	79.39	2 violations: MW >500, MLOGP >4.15	CYP2D6
MLK_MOD-44	583.78	5.65	4	2	107.63	2 violations: MW >500, MLOGP >4.15	CYP2C19, CYP2D6, CYP3A4
MLK_MOD-45	567.71	4.84	5	2	92.53	2 violations: MW >500, MLOGP >4.15	CYP2C19, CYP2D6, CYP3A4
MLK_MOD-46	578.74	4.98	5	2	92.28	2 violations: MW >500, MLOGP >4.15	CYP2C19, CYP2D6, CYP3A4
MLK_MOD-47	553.69	4.35	6	2	105.17	2 violations: MW >500, MLOGP >4.15	CYP2C19, CYP2C9, CYP2D6, CYP3A4
MLK_MOD-48	541.68	4.47	5	3	108.07	2 violations: MW >500, MLOGP >4.15	CYP2C9, CYP2D6, CYP3A4

MLK_MOD-49	645.78	5.72	6	2	109.60	2 violations: MW >500, MLOGP >4.15	CYP2D6
MLK_MOD-50	619.79	5.50	5	2	88.62	2 violations: MW >500, MLOGP >4.15	CYP2C9, CYP3A4
