# Supplementary material for: Computational Investigation of Montelukast and Its Structural Derivatives for Binding Affinity to Dopaminergic and Serotonergic Receptors: Insights from a Comprehensive Molecular Simulation
Source: Pharmaceuticals (Basel). 2025 Apr 10;18(4):559. doi: 10.3390/ph18040559 (PMC12030116; doi:10.3390/ph18040559)
Supplement: Supplementary file 1 [file pharmaceuticals-18-00559-s001.zip › Supplementary Data S5 - Predicted drug-likeness and toxicity profiles of Montelukast derivatives.pdf]

SUPPLEMENTARY DATA S5

IN SILICO DRUG-LIKENESS AND TOXICITY EVALUATION

Molecule	Drug-likeness	Mutagenic	Tumorigenic	Reproductive Effective	Irritant
MLK_MOD-1	1.433	None	None	High	None
MLK_MOD-2	1.433	None	None	High	None
MLK_MOD-3	0.093	None	None	High	None
MLK_MOD-4	1.433	None	None	None	None
MLK_MOD-5	2.958	None	None	None	None
MLK_MOD-6	2.669	High	None	None	None
MLK_MOD-7	1.648	None	None	None	None
MLK_MOD-8	2.632	None	None	High	None
MLK_MOD-9	2.224	None	None	High	None
MLK_MOD-10	2.376	None	None	High	None
MLK_MOD-11	-11.864	None	None	High	None
MLK_MOD-12	1.433	None	None	High	None
MLK_MOD-13	0.093	None	None	High	None
MLK_MOD-14	1.523	None	None	High	None
MLK_MOD-15	1.569	None	None	High	None
MLK_MOD-16	-5.756	None	None	High	None
MLK_MOD-17	6.137	None	None	High	None
MLK_MOD-18	-11.864	None	None	High	None
MLK_MOD-19	3.251	None	None	High	None
MLK_MOD-20	1.582	None	None	High	None
MLK_MOD-21	2.031	None	None	None	None
MLK_MOD-22	1.433	None	None	None	None
MLK_MOD-23	1.433	None	High	High	None
MLK_MOD-24	1.433	None	High	High	None
MLK_MOD-25	1.918	Low	Low	High	None
MLK_MOD-26	0.373	High	High	High	None
MLK_MOD-27	-5.765	None	High	High	None
MLK_MOD-28	-0.356	None	None	High	None
MLK_MOD-29	1.183	None	None	High	None
MLK_MOD-30	1.351	None	None	High	None
MLK_MOD-31	-0.869	None	None	High	None
MLK_MOD-32	1.509	None	None	High	None
MLK_MOD-33	-0.036	None	None	High	High
MLK_MOD-34	4.236	None	None	High	None
MLK_MOD-35	3.371	None	None	High	High
MLK_MOD-36	1.504	None	None	High	None
MLK_MOD-37	-0.411	None	None	High	None
MLK_MOD-38	-5.077	None	None	High	None
MLK_MOD-39	-0.862	Low	Low	High	None
MLK_MOD-40	-18.521	None	None	High	High
MLK_MOD-41	1.433	None	None	High	None
MLK_MOD-42	4.862	None	None	High	None
MLK_MOD-43	1.433	None	None	High	None
MLK_MOD-44	3.182	None	None	High	None
MLK_MOD-45	1.648	None	None	High	None

MLK_MOD-46	1.433	None	None	High	None
MLK_MOD-47	1.433	None	None	High	None
MLK_MOD-48	1.882	Low	Low	High	None
MLK_MOD-49	1.417	Low	Low	High	None
MLK_MOD-50	0.070	Low	Low	High	None
